# Exposure of iPSC-derived human microglia to brain substrates enables the generation and manipulation of diverse transcriptional states in vitro

**DOI:** 10.1038/s41590-023-01558-2

**Published:** 2023-07-27

**Authors:** Michael-John Dolan, Martine Therrien, Saša Jereb, Tushar Kamath, Vahid Gazestani, Trevor Atkeson, Samuel E. Marsh, Aleksandrina Goeva, Neal M. Lojek, Sarah Murphy, Cassandra M. White, Julia Joung, Bingxu Liu, Francesco Limone, Kevin Eggan, Nir Hacohen, Bradley E. Bernstein, Christopher K. Glass, Ville Leinonen, Mathew Blurton-Jones, Feng Zhang, Charles B. Epstein, Evan Z. Macosko, Beth Stevens

**Affiliations:** 1 Stanley Center for Psychiatric Research, The Broad Institute of MIT and Harvard, Cambridge, MA USA; 2 Boston Children’s Hospital, F.M. Kirby Neurobiology Center, Boston, MA USA; 3 Harvard Medical School, Boston, MA USA; 4 Broad Institute of MIT and Harvard Cambridge, Cambridge, MA USA; 5 Department of Biological Engineering, MIT, Cambridge, MA USA; 6 Howard Hughes Medical Institute, Boston, MA USA; 7 Department of Brain and Cognitive Science, MIT, Cambridge, MA USA; 8 McGovern Institute for Brain Research at MIT, Cambridge, MA USA; 9 Department of Stem Cell and Regenerative Biology, Harvard University, Cambridge, MA USA; 10 Leiden University Medical Center, LUMC, Leiden, the Netherlands; 11 Department of Medicine, Center for Cancer Research, Massachusetts General Hospital, Boston, MA USA; 12 Department of Medicine, Harvard Medical School, Boston, MA USA; 13 Department of Cancer Biology, Dana-Farber Cancer Institute, Boston, MA USA; 14 Ludwig Center at Harvard, Harvard Medical School, Boston, MA USA; 15 Departments of Cell Biology and Pathology, Harvard Medical School, Boston, MA USA; 16 Department of Cellular and Molecular Medicine, University of California San Diego, La Jolla, CA USA; 17 Department of Neurosurgery, Kuopio University Hospital and Institute of Clinical Medicine – Neurosurgery, University of Eastern Finland, Kuopio, Finland; 18 Department of Neurobiology and Behavior, Sue and Bill Gross Stem Cell Research Center, UCI Institute for Memory Impairments and Neurological Disorders, Institute for Immunology, University of California, Irvine, CA USA; 19 Massachusetts General Hospital, Department of Psychiatry, Boston, MA USA

**Keywords:** Neuroimmunology, Inflammation

## Abstract

Microglia, the macrophages of the brain parenchyma, are key players in neurodegenerative diseases such as Alzheimer’s disease. These cells adopt distinct transcriptional subtypes known as states. Understanding state function, especially in human microglia, has been elusive owing to a lack of tools to model and manipulate these cells. Here, we developed a platform for modeling human microglia transcriptional states in vitro. We found that exposure of human stem-cell-differentiated microglia to synaptosomes, myelin debris, apoptotic neurons or synthetic amyloid-beta fibrils generated transcriptional diversity that mapped to gene signatures identified in human brain microglia, including disease-associated microglia, a state enriched in neurodegenerative diseases. Using a new lentiviral approach, we demonstrated that the transcription factor MITF drives a disease-associated transcriptional signature and a highly phagocytic state. Together, these tools enable the manipulation and functional interrogation of human microglial states in both homeostatic and disease-relevant contexts.

## Abstract (web summary)

Stevens and colleagues show that human stem-cell-differentiated microglia can be used to model the extensive transcriptional diversity of human brain microglia.

## Main

Microglia, the macrophages of the brain, have central roles in development, homeostasis and diseases of the central nervous system (CNS)^1^. Genetic studies have implicated microglia in late-onset Alzheimer’s disease (AD), with a large fraction of AD risk genes expressed in myeloid cells^2,3^. Moreover, microglia activation and dysfunction are hallmarks of AD and other neurodegenerative disorders^4^. Single-cell RNA sequencing (scRNA-seq) has revealed that microglia exist in multiple states^5–14^ that each express distinct gene signatures, including disease-associated microglia (DAMs). However, the function of these states and their impact on disease remain unknown.

As the differential expression of many genes defines microglial states, high-throughput models are needed to understand how transcriptional signatures map onto microglial function. However, there are several outstanding challenges. Many differences exist between mouse and human microglia^15^, a particularly acute problem with AD risk genes^16^. Microglia also rapidly alter their gene expression upon primary culture, presumably owing to a lack of brain-derived cues^15,17^, and cell lines fail to accurately model this cell type^18^. Finally, high-throughput gene perturbation studies are not possible as microglia are resistant to conventional forms of DNA delivery^19^.

Here, we addressed these issues using human stem-cell-differentiated microglia (iMGLs) by creating an in vitro platform for studying human microglial states. Exposure of iMGLs to brain-related substrates, scRNA-seq and cross-dataset integration showed that transcriptional states identified in the human brain could be recapitulated in a monolayer culture. In particular, we identified DAM-like states in vitro and demonstrated their formation was dependent on signaling through cell surface receptor TREM2, as observed in vivo^6,20^. Using a new lentiviral transduction protocol that opens up iMGLs to efficient viral manipulation, we determined that the transcription factor MITF regulates disease-associated genes and a highly phagocytic state. Finally, we demonstrated that our observations were robust by differentiating iMGLs from multiple human induced pluripotent stem cell (iPSC) lines.

## Results

### CNS substrates induce transcriptional states in iMGLs

**Fig. 1:**
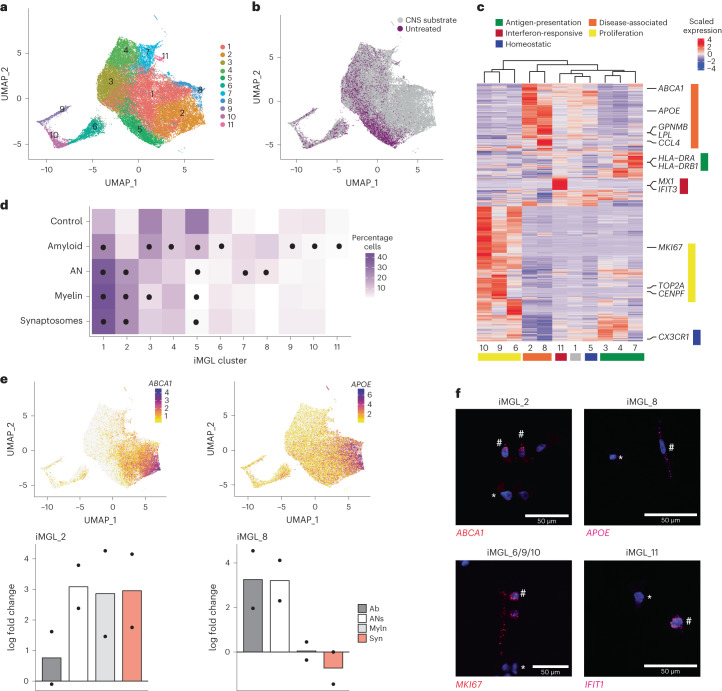
Treatment of iMGLs with CNS substrates induces diverse transcriptional states that map to those found in vivo. **a**, Uniform manifold approximation and projection (UMAP) of iMGLs that were either untreated or treated for 24 h with synaptosomes, myelin debris, synthetic Aβ fibrils or ANs (collectively referred to as CNS substrates) followed by scRNA-seq; total of 56,454 cells across two replicates, cells colored by cluster. **b**, UMAP projection as in **a**; cells colored as untreated or CNS-substrate-treated condition. **c**, Heatmap of differentially enriched genes for each cluster (iMGL_1-11) sorted by similarity and microglial states; states are labeled. **d**, Mean relative abundance of each cluster across each condition. Circles represent significant differences (adjusted *P* < 0.05; Supplementary Table 3) determined by a Dirichlet regression test for differential abundance. **e**, Marker gene expression (top) and log fold change of cluster relative to untreated (bottom) for clusters iMGL_2 (left) and iMGL_8 (right) (*n* = 2). Syn, synaptosomes; Myln, myelin debris; Ab, synthetic Aβ fibrils. **f**, Representative images of gene expression with fluorescent in situ hybridization for disease-associated (iMGL_2 and iMGL_8), proliferation (iMGL_6/9/10) and interferon-responsive (iMGL_11) states. All cells were positive for expression of *C1QA*; not shown for clarity. The hash symbol indicates a positive cell and the asterisk indicates a negative cell. Scale bar, 50 μm. See Extended Data Fig. 4 for quantifications per condition.

**Extended Data Fig. 1:**
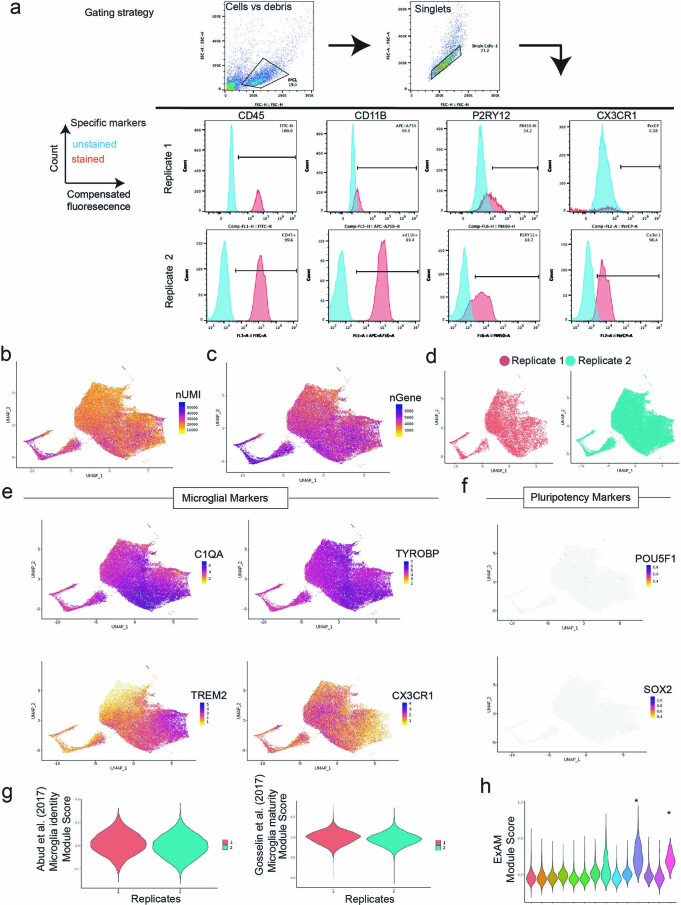
Single-cell RNAseq of iMGLs and quality control. a) Flow cytometry gating strategy and plots illustrating expression of microglial protein markers from the two independent differentiations used for the scRNAseq dataset. Cells underwent either flow cytometry or single-cell RNAseq. b-c) UMAP projection (as in Fig. 1a) of quality control metrics for iMGL dataset highlighting the number of unique molecular identifiers (nUMI, b) and the number of genes (nGene, c). d) UMAP projection (as in Fig. 1a), cells colored by replicate. e) UMAP projection (as in Fig. 1a) showing expression of four key microglial markers. f) UMAP projection (as in Fig. 1a) showing expression of two stem cell markers. g) Module scores for microglia identity and maturity per cell, plotted by replicate h) Module scores for microgliaExAM signature^22^ per cells plotted per iMGL cluster. Two high ExAM clusters were removed(*). Note n = 2 independent differentiations for B-F.

Microglia cultured in vitro lack key transcriptional features observed in vivo^15,17^. To test whether exposure to complex CNS substrates drives the formation of microglial states in a dish, we differentiated human embryonic stem cells (H1) into iMGLs^21^ and, at day 40, performed scRNA-seq on untreated iMGLs or cells incubated with synaptosomes, myelin debris, apoptotic neurons (ANs) or synthetic amyloid-beta (Aβ) fibrils for 24 h. We acquired a total of 56,454 single iMGL transcriptomes across all conditions (referred subsequently to as the iMGL dataset) (Fig. 1a, Extended Data Fig. 1a–d and Supplementary Table 1). Cells in both replicates showed minimal expression of pluripotency markers and an enrichment of microglial identity and maturity signatures (Extended Data Fig. 1e–g), confirming successful differentiation. We excluded 2.4% of the iMGL population (Extended Data Fig. 1h), as these cells showed an artifactual gene signature known to be induced by handling in single-cell preparations^22^.

**Extended Data Fig. 2:**
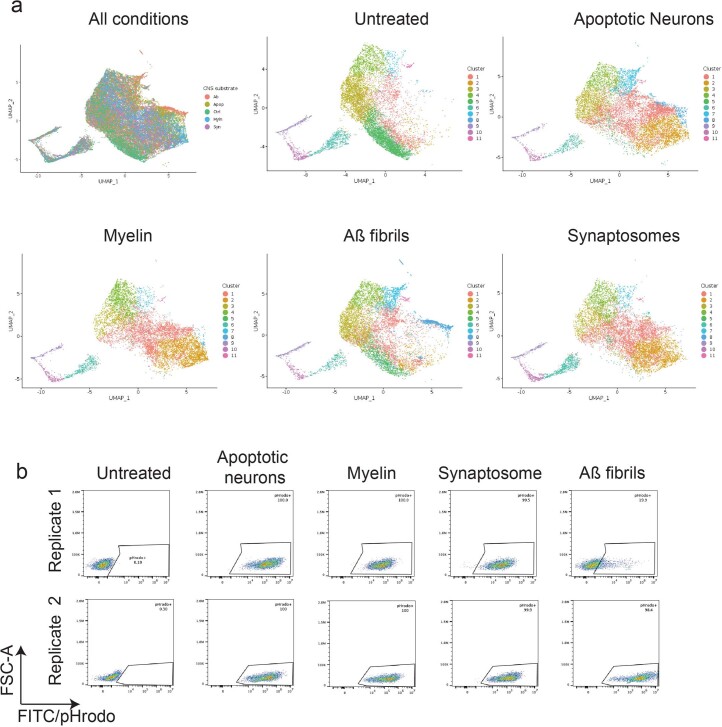
Single-cell RNAseq data and phagocytosis of substrates for each condition. **a)** UMAP projection of iMGL dataset for each condition (n = 2 independent differentiations per condition), cells are colored by conditions. b) Flow cytometry measurement of pHrodo-488/FITC for each CNS substrate exposed to the iMGL dataset. FSC=forward scatter. FITC/pHrodo=phagocytosis of substrate.

Applying unbiased clustering to all conditions combined, we identified 11 clusters (clusters iMGL_1 to iMGL_11) of iMGLs, which were detected across replicates (Fig. 1a and Extended Data Fig. 1d), indicating a degree of transcriptional diversity similar to that reported in human postmortem datasets^11,23–25^. We observed that exposure to synaptosomes, myelin debris, ANs or Aβ fibrils induced a shift in iMGL transcriptional signature (Fig. 1b and Extended Data Fig. 2a). Although all substrates induced phagocytosis in iMGLs (Extended Data Fig. 2b), not all treated iMGLs adopted the same states in response to the treatment (Fig. 1c,d). This indicates that iMGLs are not a homogeneous population but form diverse transcriptional states in response to different CNS substrates, as observed in vivo^1^.

**Extended Data Fig. 3:**
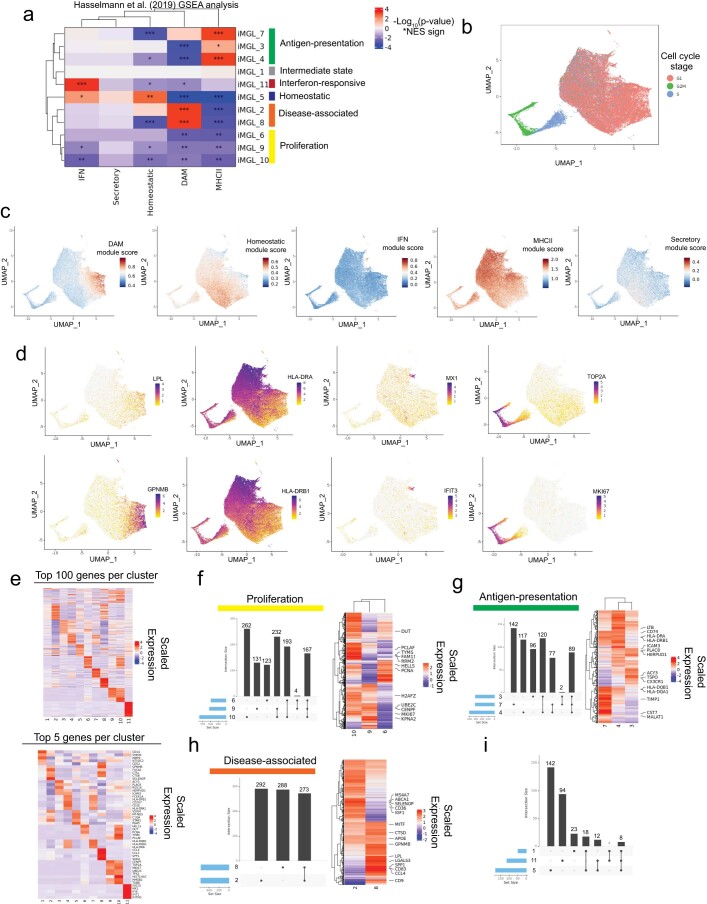
Annotation and gene expression signatures of iMGL datasets. A) Heatmap illustrating the relative GSEA enrichment significance of positively enriched marker genes from each microglial cluster from xenotransplanted iMGLs *in vivo*^26^, within all differentially expressed genes for each iMGL cluster. b) UMAP projection of iMGL dataset, cells colored by cell cycle phase (G1, G2M and S) c) UMAP projection of iMGL dataset, cells colored by module scores of transcriptional signature identified in xenotransplanted iMGLs *in vivo*^26^. d) UMAP projection of iMGL dataset, cells colored by expression of *LPL*, *GPNMB* (iMGL_2 and iMGL_8), *HLA-DRA, HLA-DRB1* (marker genes for iMGL_3, iMGL_4 and iMGL_7), *MX1*, *IFIT3* (marker genes for iMGL_11), *TOP2A* or *MKI67* (marker genes for iMGL_6, iMGL_9, iMGL_10). e) Heatmap of the top 100 (top) or top 5 (bottom) differentially expressed genes for each iMGL cluster. f-h) Upset plots (left) summarizing the number of unique and shared differentially expressed genes for each cluster within each microglial state identified and heatmaps (right) showing the expression of positively enriched genes for the constituent clusters of each state. i) Upset plots (as in g) for clusters iMGL_1, iMGL_5 and iMGL_11.

**Extended Data Fig. 4:**
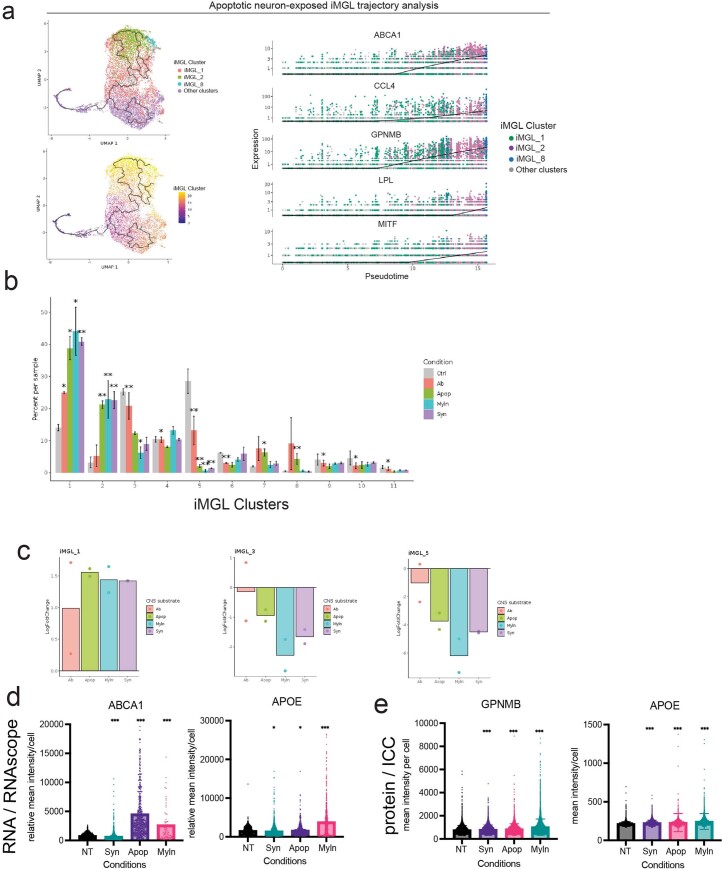
iMGL clusters by trajectory, substrate exposure and validation with in situ hybridization and ICC. a) Single cell profiles of apoptotic neuron-exposed iMGLs (n = 2) in UMAP space (left) and ordered by pseudotime (right) for clusters iMGL_1, iMGL_2 and iMGL_8. b) Barchart of percentage composition of cells per condition per cluster. Statistical significance determined by Dirichlet regression. * = p-value < 0.05 and ** = p-value < 0.01. c) Fold change of iMGL_1,iMGL_2 and iMGL_4 relative to the untreated control condition d) Quantification of fluorescent *in situ* hybridization of *ABCA1* and *APOE* by mean intensity per cell. *ABCA1*: NT vs Syn p < 0.0001, NT vs Apop p < 0.0001, NT vs Myln p < 0.0001. *APOE*: NT vs Syn p = 0.076, NT vs Apop p = 0.01786, NT vs Myln p < 0.0001. e) Quantification immunocytochemistry of *GPNMB* and *APOE* by mean intensity per cell. *GPNMB*: NT vs Syn p < 0.001, NT vs Apop p < 0.0001, NT vs Myln p < 0.0001. *APOE*: NT vs Syn < 0.001, NT vs Apop p < 0.0001, NT vs Myln <0.0001. For d-e) >500 cells were counted by conditions across 4 biological replicates. NT= Not treated, Syn=synaptosomes, Myln=myelin debris, Ab=synthetic Aβ fibrils, Apop= apoptotic neurons.

To understand the biological meaning of the 11 clusters observed, we performed a differential gene expression analysis (Fig. 1c and Supplementary Table 2). By leveraging previously published data^26^, we classified each of the 11 clusters into established microglial states (Extended Data Fig. 3a–c)^27^ and identified iMGL states similar to those identified in vivo. This included a neurodegenerative DAM state (clusters iMGL_2 and iMGL_8, based on the expression of *APOE*, *GPNMB*, *LPL* and *ABCA1*), an antigen-presenting state (clusters iMGL_3, iMGL_4 and iMGL_7, expressing *HLA-DRA* and *HLA-DRB1*), an interferon-responsive state (cluster iMGL_11, *IFIT3* and *MX1*), a proliferation state (clusters iMGL_6, iMGL_9 and iMGL_10, *MKI67* and *TOP2A*) and a homeostatic state (iMGL_5, *CX3CR1*) (Fig. 1c–e and Extended Data Fig. 3d,e). To validate the presence of these clusters, we treated iMGL with CNS substrates for 24 h and performed in situ hybridization to detect key markers of microglial states (*ABCA1* for iMGL_2, *APOE* for iMGL_8, *MKI67* for iMGL_6/9/10 and *IFIT1* for iMGL_11) (Fig. 1f), confirming that expression of these markers was present or increased after treatment. Three of these states (proliferation, antigen-presenting and DAM) consisted of multiple clusters, each expressing a large number of both unique and shared differentially expressed genes (Extended Data Fig. 3f–i). In addition, a pseudotime analysis^28^ indicated that one cluster, iMGL_1, appeared to be in transition from a homeostatic to a disease-associated state (iMGL_1 to iMGL_2 and iMGL_8) (Extended Data Fig. 4a), reflecting ongoing microglial dynamics 24 h after stimulation. Thus, our data suggest that iMGLs adopt diverse transcriptional states in vitro after treatment with CNS substrates.

We next considered whether exposing iMGLs to CNS substrates resulted in context-dependent cell states. We observed many statistically significant changes in the proportions of iMGL clusters after exposure to CNS substrates (Fig. 1d, Extended Data Fig. 4b and Supplementary Table 3). Several clusters changed similarly across multiple conditions. For example, iMGL_5 was depleted in response to all treatments compared with no treatment, whereas iMLG_1 was enriched in all treated iMGLs compared with untreated iMGL (Fig. 1d and Extended Data Fig. 4c). Other clusters had substrate-specific responses; for instance, cluster iMGL_2 was broadly induced by all substrates except Aβ fibrils, whereas iMGL_8 was only induced by ANs and Aβ fibrils (Fig. 1d,e), although the change was statistically significant only in the former case. The application of in situ hybridization and immunocytochemistry for iMGL_2 and iMGL_8 cluster-enriched markers (*APOE*, *GPNMB* and *ABCA1*) (Extended Data Fig. 4d,e), indicated enrichment of these signatures in response to ANs and several other substrates. These results confirm that iMGLs form distinct cell states in response to specific CNS stimuli.

**Fig. 2:**
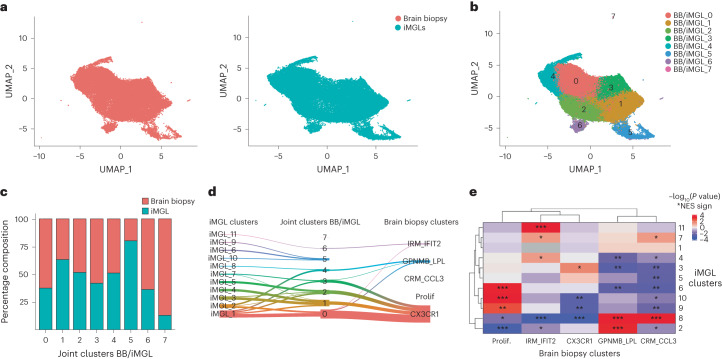
Dataset integration of iMGL and human cortical biopsy microglia reveals analogous transcriptional states. **a**, UMAP projection of integrated human brain biopsy microglia (left) and iMGL profiles (right). Cells are colored by dataset. **b**, UMAP projection of integrated human brain biopsy microglia and iMGL profiles. Cells are colored by BB/iMGL joint cluster. **c**, Proportion of cells per joint cluster from each source of data (human brain biopsy microglia or iMGLs), normalized by the proportion of total cells per dataset. **d**, River plot showing the relationship between joint clusters and the clusters defined in both the iMGL and brain biopsy datasets. Links with fewer than 200 cells have been removed for clarity. **e**, Heatmap illustrating the relative enrichment significance (as determined by fgsea; Methods) of positively enriched marker genes from each human brain biopsy cluster, within all differentially expressed genes for each iMGL cluster. Prolif, proliferative; NES, normalized enrichment score. *P < 0.05, **P < 0.01, ***P < 0.001.

**Extended Data Fig. 5:**
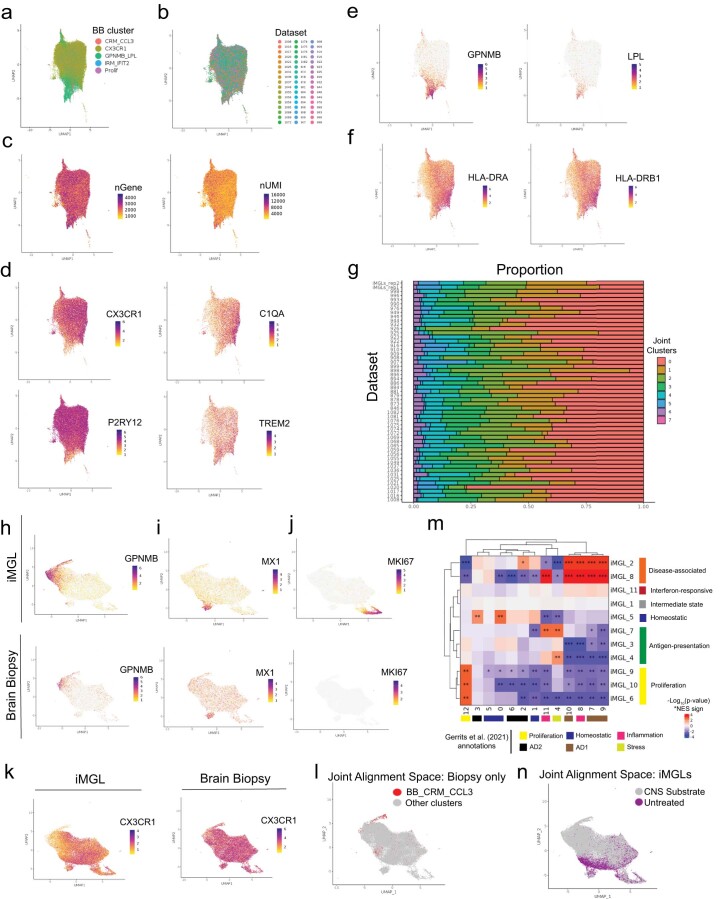
Summary of the human cortical biopsy microglial dataset and LIGER alignment with iMGL dataset. a) UMAP projection of cluster annotations of human brain biopsy (BB dataset) single dataset from ref. ^29^. b) UMAP projection, cells colored by patient. c) UMAP projection (as in a) number of genes (nGene, left) and number of unique molecular identifiers (nUMI, right). d-f) UMAP projection (as in a), cells colored by expression of *CX3CR1*, *C1QA*, P2RY12, *TREM2* (d), *GPNMB* and *LPL* (e), *HLA-DRA* and *HLA-DRB1* (f*)*. g) Proportion of cells across joint clusters for each dataset (iMGL or brain biopsy dataset). h-k) UMAP projection LIGER integration of iMGL or brain biopsy dataset, cells colored by datasets (iMGL top, brain biopsy bottom) for expression of *GPNMB* (h), *MX1* (i), *MKI67* (j), *CX3CR1* (k, iMGL left, brain biopsy right). l) UMAP projection (as in h), brain biopsy cells only with the BB_CRM_CCL3 state highlighted. m) Heatmap of the relative enrichment significance of positively enriched marker genes from each *in vivo* human microglial cluster from ref. ^24^, within differentially expressed genes of each iMGL cluster (GSEA analysis). n) UMAP projection (as in h) of cells from iMGL dataset only colored by substrates or untreated.

Next, we systematically compared the iMGL transcriptional states with those found in vivo, using a single-nucleus RNA sequencing (snRNA-seq) dataset of microglia from human cortical brain biopsies^29^. This dataset consisted of 54,475 single microglial nuclei from 51 individuals, classified into five microglial clusters (referred as brain biopsy (BB) clusters; Extended Data Fig. 5a–f). Integrative analysis of the brain biopsy microglia with iMGL profiles using LIGER^30^ showed significant alignment between the two datasets (Fig. 2a). To quantify the alignment, we calculated for each cell the degree to which its neighbors were from one (a score of 0) or all datasets (a score of 1)^30^. This analysis resulted in an alignment score of 0.77 (Methods), suggesting extensive mixing between brain biopsy microglia and iMGL profiles. Analysis of the joint clusters generated by the LIGER integration, which we refer to as BB/iMGL, identified eight joint clusters (BB/iMGL_0 to BB/iMGL_7) (Fig. 2b), all of which were populated by both biopsy microglia and iMGLs (Fig. 2c and Extended Data Fig. 5g). Brain biopsy microglia and iMGLs contributed roughly equally to the most joint clusters (Fig. 2c), with two exceptions: BB/iMLG_5, a proliferative cluster dominated by iMGLs; and BB/iMLG_7, a small number of human-brain-specific microglia that highly expressed interferon genes but were distinct from BB/iMLG_6, the interferon-responsive joint cluster present in both datasets (Fig. 2c,d). Joint clusters mapped to established microglial states in both the iMGL and biopsy data (Fig. 2d and Extended Data Fig. 5h–k); these included BB/iMLG_4, which contained the majority of DAMs from both the iMGL (iMGL_2 and iMGL_8) and brain biopsy (BB_GPNMB_LPL) datasets (Fig. 2c,d). Joint cluster BB/iMGL_4 also contained cells from the BB_CRM_CCL3 cluster, although there were few microglia in this state (Extended Data Fig. 5l); larger datasets are needed to properly resolve such rare states^31^. Several iMGL clusters, including the homeostatic (iMGL_5) and antigen-presenting (iMGL_3, iMGL_4 and iMGL_7) states, coclustered with microglia identified as homeostatic in the brain biopsy dataset (BB_CX3CR1). We identified a gradient of antigen-presenting genes in human brain microglia from the BB_CX3CR1 cluster (Extended Data Fig. 5f), consistent with previously reported antigen-presenting states in human postmortem datasets^11,27,32^, suggesting that homeostatic microglia exhibit transcriptional diversity that can be modeled in vitro. To further validate the similarity between microglial states in substrate-exposed iMGL with human states, we performed gene set enrichment analysis (GSEA)^33^ between each iMGL cluster and clusters found in the brain biopsy dataset^29^ or a postmortem human brain microglia dataset^24^. This analysis indicated that each iMGL state significantly overlapped with those identified in the brain biopsy dataset used above (Fig. 2e) and in the microglia dataset from ref. ^24^ (Extended Data Fig. 5m). Finally, we found that iMGLs exposed to substrates overlapped with some of these disease-associated states (Extended Data Fig. 5n). Together, these findings indicate that substrate-exposed iMGLs can exhibit transcriptional profiles analogous to those found in human brain microglia^27^.

### Human DAM state can be induced in vitro

**Fig. 3:**
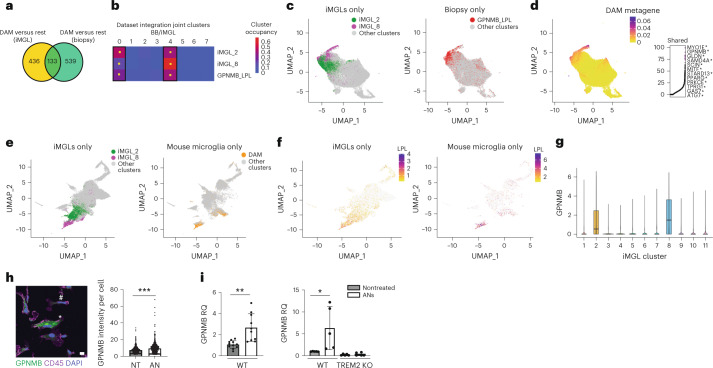
DAMs generated in vitro are similar to those found in human and in mouse in vivo models. **a**, Venn diagram showing overlap between DAM signature iMGL and brain biopsy (*P* = 3.06 × 10^−68^, hypergeometric test). **b**, Percentage distribution of cells from iMGL_2, iMGL_8 and BB_GPNMB_LPL over joint clusters BB/iMGL. Yellow square indicates significant enrichment using binomial test, *P* < 0.05. **c**, UMAP projection (as in Fig. 2) colored by cluster identity. **d**, UMAP projection (as in **c**) showing the metagene common to both iMGL and brain biopsy DAMs (left) and top genes (right). Asterisk labels genes in the ‘AD1’ DAM cells identified in ref. ^24^. **e**, UMAP projection of LIGER integration of iMGL and microglia from the 5xFAD mouse model, cells colored by cluster: iMGL_2 (green), iMGL_8 (magenta) or mouse microglia with DAMs (orange). **f**, UMAP projection (as in **e**) showing expression of *LPL* in iMGLs (left) and mouse microglia (right). **g**, Violin plot of *GPNMB* mRNA expression plotted by iMGL cluster; *n* = 2 replicates per condition, all five conditions pooled. Boxes show the first and third quartiles of the data with a line marking the median. Whiskers mark values closest to 1.5 times the interquartile range; no outliers are plotted. **h**, Immunocytochemistry of GPNMB expression in iMGLs treated with ANs or PBS. Left, representative image showing GPNMB (green) and CD45 (magenta, microglia marker). Asterisk indicates a positive cell, and hash indicates a negative cell. Right, quantitative analysis (two-tailed *t*-test, ***P < 0.001; at least 500 cells were counted per condition, data combined from four replicates, mean and s.d. are shown). Scale bar, 50 μm. **i**, Quantitative rtPCR of *GPNMB* expression in H1 iMGLs (left) and TREM2-deficient or isogenic iMGLs (right) treated with ANs or PBS. For H1 iMGL, two-tailed *t*-test, *P* = 0.0009, *n* > 4, mean and s.d. of relative quantification (RQ) are shown; for TREM2-deficient or isogenic iMGL, two-tailed *t*-test, *P* = 0.0427, *n* > 4, mean and s.d. are shown. KO, knockout; WT, wild type. *P < 0.05, **P < 0.01.

**Extended Data Fig. 6:**
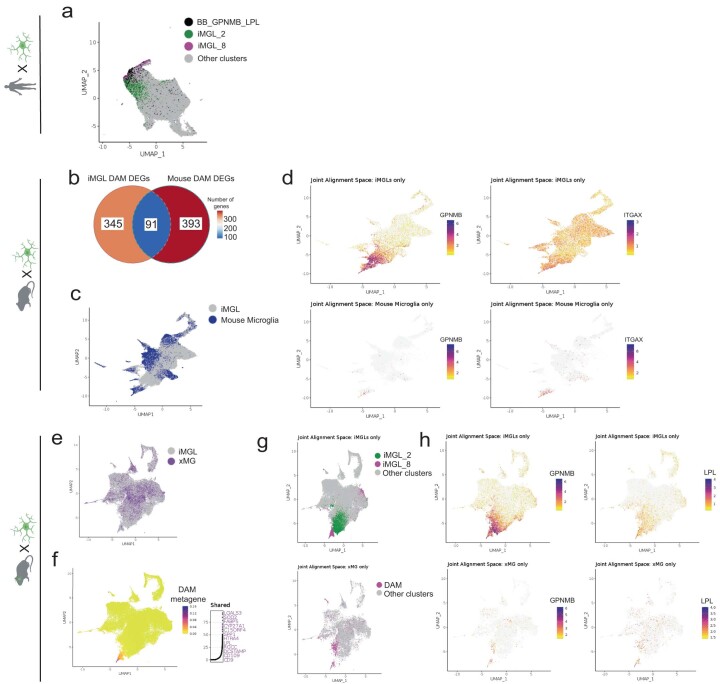
Dataset integration of iMGLs with mouse AD and xenotransplanted human microglia. a) UMAP projection of LIGER integration of iMGL or brain biopsy dataset, cells colored by identity to cluster iMGL_2 (green), iMGL_8 (magenta) highlighted or BB_GPNMB_LPL cluster (black). b) Comparing overlap of iMGL DAM (iMGL_2 and iMGL_8) enriched genes with those enriched in murine DAMs in the 5xFAD genotype^6^ (p value: 5.41e-19, hypergeometric test). c) UMAP projection of LIGER integration of iMGL datasets with *in vivo* mouse microglia (from wild-type and 5xFAD genotypes). Cells colored by dataset. d) UMAP projection (as in c) of iMGLs (top) or mouse microglia (bottom) cells colored for expression of *GPNMB* (left) and *ITGAX* (right). e) UMAP projection LIGER integration of iMGLs and xenotransplanted iMGLs. Cells colored by dataset. f) UMAP projection (as in e) colored by expression of shared factor DAM metagene. Right, top constituent genes of this shared factor. g) UMAP projection (as in e), of iMGLs (top) or xenograft microglia (bottom) colored by identity to iMGL_2, iMGL_8 or DAM. h) UMAP projection (as in e), of iMGLs (top) or xenograft microglia (bottom) cells colored for expression of *GPNMB* (left) and *LPL* (right).

DAMs are enriched in both human patients with AD^23,24^ and mouse models of neurodegeneration^6,34^, and this state depends on TREM2 receptor signaling^6,20,25,34^. To further examine the generation of DAM-like iMGL clusters using our in vitro platform, we first compared the gene expression signatures of DAM-like iMGLs with the DAM signature observed in the brain biopsy dataset. We compiled a list of DAM-associated genes by comparing DAM-like iMGLs (iMGL_2 and cluster iMGL_8 versus all other iMGL clusters) or human brain biopsy DAMs (BB_GPNMB_LPL versus all other brain biopsy clusters) with all the other clusters from the respective datasets. Comparison of these gene lists indicated a significant shared transcriptional signature between iMGL and brain biopsy DAMs (Fig. 3a). These shared genes included *ABCA1*, *APOE*, *GPNMB*, *LPL* and *TREM2* (Supplementary Table 4), confirming our GSEA results in which iMGL_2 and iMGL_8 were enriched for genes found in brain biopsy DAMs (BB_GPNMB_LPL) (Fig. 2e). To further evaluate the similarities between in vivo and iMGL DAMs, we examined the iMGL and brain biopsy microglia dataset integration. We observed that iMGL_2 and iMGL_8 cells were statistically significantly enriched in the same joint clusters (BB/iMGL_0 and BB/iMGL_4) as in vivo DAMs (BB_GPNMB_LPL) (Fig. 3b–d and Extended Data Fig. 6a). These cells, regardless of dataset source, shared a transcriptional signature (LIGER metagene) enriched for genes involved in neurodegeneration and amyloid pathology, including *GPNMB*, *MYO1E, ABCA1*, *CD9* and *APOE* (Fig. 3d and Supplementary Table 5)^6,23,24^. These results indicate that CNS substrate exposure can induce a DAM transcriptional state in vitro.

To understand the role of pathology in the formation of the DAM state, we integrated iMGL single-cell profiles with single-cell transcriptomic datasets from mouse^6^ or xenotransplanted human microglia^26^ exposed to amyloid plaques in an AD mouse model (5xFAD). Dataset integration indicated alignment of iMGL DAMs with 5xFAD mice microglia (Fig. 3e–g and Extended Data Fig. 6b–d, alignment score 0.58) and 5xFAD xenotransplanted iMGLs (Extended Data Fig. 6e–h, alignment score 0.83). We observed colocalization of iMGL DAMs, especially iMGL_8, with mouse DAMs and demonstrated significant overlap in gene signature using a hypergeometric test (Extended Data Fig. 6b). We also observed colocalization and shared gene signatures of iMGL_2 and iMGL_8 with xenotransplanted DAMs (Extended Data Fig. 6e–h). These results indicate a key role for amyloid pathology in DAM formation.

Mouse and human DAM transcriptomic profiles fall on a continuum^6,24^. We ordered the AN-exposed iMGLs, which included both iMGL DAM clusters (12,217 cells, *n* = 2), on a pseudotime trajectory based on their transcriptional profiles^28^. This showed a transition from cluster iMGL_1 to iMGL_2 to iMGL_8 (Extended Data Fig. 4a), suggesting that CNS substrate stimuli differentially drove iMGLs along a disease-associated trajectory, explaining the existence of multiple DAM-like iMGL clusters.

**Extended Data Fig. 7:**
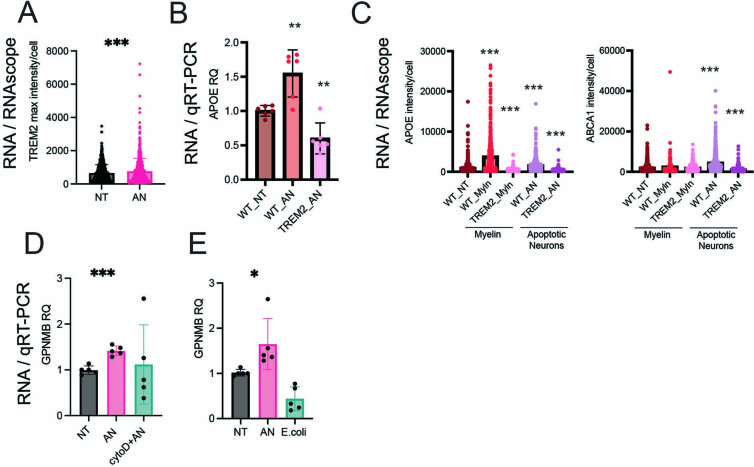
Dependence of iMGL DAM formation on TREM2 expression and substrate. a) Quantification of relative intensity of TREM2 antibody stain determined by immunocytochemistry (p-value < 0.0001). b) rtPCR of APOE expression in WT and TREM2 KO iMGL(APOE WT_NT vs WT_AN p-value < 0.0035, APOE WT_AN vs TREM2_AN p-value < 0.0002). ABCA1 WT_NT vs WT_MYLN p-value = 0.074, c) APOE, ABCA1 expression level measured by RNAscope. (APOE WT_NT vs WT_MYLN p-value < 0.0001, APOE WT_MYLN vs TREM2_MYLN p-value < 0.0001, APOE WT_NT vs WT_AN p-value < 0.0001, APOE WT_AN vs TREM2_AN p-value < 0.0001, ABCA1 WT_NT vs WT_MYLN p-value = 0.0740, ABCA1 WT_MYLN vs TREM2_MYLN p-value = 0.0754, ABCA1 WT_NT vs WT_AN p-value < 0.0001, ABCA1 WT_AN vs TREM2_AN p-value < 0.0001). For b-c: At least 500 cells were counted by conditions across 4 biological replicates d) rtPCR of GPNMB mRNA in iMGLs untreated or exposed to AN or AN+ cytochalasin D (p-value < 0.0001) e) rtPCR of GPNMB mRNA in iMGLs untreated, treated with AN or E. coli (p-value = 0.0374).

Mouse and human microglia depend on TREM2 receptor signaling to induce DAM signatures^6,20,34^. *TREM2* messenger RNA (mRNA) was highly expressed in iMGL_2 and iMGL_8 (Extended Data Fig. 1e), and TREM2 protein expression was elevated in iMGLs treated with ANs (Extended Data Fig. 7a). Glycoprotein NMB (*GPNMB*) specifically labeled both iMGL_2 and iMGL_8 DAM clusters (Fig. 3g) and was also enriched in BB_GPNMB_LPL microglia in the human biopsy data (Fig. 3d and Extended Data Fig. 5e) and published postmortem studies^6,20,23,24,26^. Immunocytochemistry and quantitative real-time PCR (rtPCR) indicated that exposure to ANs drove an increase in GPNMB in iMGLs compared with untreated cells (Fig. 3h,i and Extended Data Fig. 4e). The AN-induced or myelin-debris-induced increase in expression of *GPNMB*, *APOE* and *ABCA1* was lost in an iMGL line that lacked TREM2 (ref. ^20^) compared with the isogenic control (Fig. 3i and Extended Data Fig. 7b,c), indicating that the DAM-like state in iMGLs is dependent on TREM2. Treatment of iMGL with ANs and cytochalasin D did not lead to induction of *GPNMB* (Extended Data Fig. 7d), indicating that the expression of *GPNMB* is dependent on phagocytosis. *GPNMB* was not induced by *Escherichia coli* (Extended Data Fig. 7e), suggesting that DAM formation is specific to CNS substrates. Together, these data suggest that iMGL DAMs share characteristics with human and mouse DAMs and are formed specifically in response to CNS-derived stimuli.

### MITF is a driver of the DAM signature and phagocytosis

**Fig. 4:**
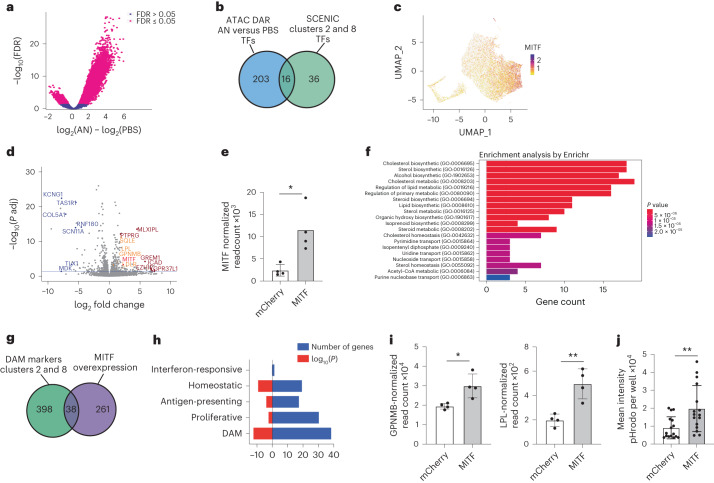
MITF is a key DAM regulator and driver of phagocytosis. **a**, Volcano plot of differentially accessible peaks according to ATAC-seq of iMGLs exposed to ANs compared with untreated. Peaks with false discovery rate (FDR) ≤ 0.05 are shown in magenta. **b**, Intersection of transcription factors nominated by ATAC-seq and SCENIC analysis. **c**, UMAP projection of iMGL dataset (as in Fig. 1a) showing *MITF* expression in iMGLs treated with CNS substrates. **d**, Volcano plot of differentially expressed genes (DEG) in the iMGLs overexpressing MITF (*n* = 4) compared with those overexpressing mCherry (*n* = 4). Blue indicates top downregulated genes, dark red indicates top upregulated genes and orange indicates genes involved in DAM signature and lipid metabolism. Blue line represents adjusted FDR ≤ 0.05. *P* adj, adjusted *P* value. **e**, Quantification of *MITF* expression in iMGLs transduced with mCherry-expressing and MITF-expressing lentivirus (two-tailed *t*-test, *P* = 0.0129, mean and s.d. are shown; *n* = 4 for each condition). **f**, Bar graph of gene ontology analysis for MITF-overexpression DEG. Bar length represents the number of genes, and shading represents statistical significance. **g**, Venn diagram of overlap between DEG in iMGL samples transduced with MITF-expressing versus mCherry-expressing lentivirus, and DEG in iMGL_02 and iMGL_8; hypergeometric test, *P* = 1.098 × 10^−12^. **h**, Comparison of MITF-overexpression genes with iMGL states; numbers of genes and *P* values from the hypergeometric test are shown. **i**, Quantification of *GPNMB* expression (left, two-tailed *t*-test, *P* = 0.0159, *P < 0.05) and *LPL* expression (right, two-tailed *t*-test, *P* = 0.0042, **P < 0.01) in RNA sequencing samples as in **d**. **j**, Mean intensity of pHrodo-conjugated AN in iMGLs transduced with either MITF or mCherry (two-tailed *t*-test, *P* = 0.0051, **P < 0.01; *n* > 5 for each condition). In all bar graphs, error bars represent s.d.

To identify transcription factors that could be key regulators of DAM states, we performed ATAC-seq (assay for transposase-accessible chromatin using sequencing) on both untreated and AN-exposed iMGLs to identify regions with changes in chromatin and in accessibility of transcription factor binding sites. Treatment with ANs increased the number of differentially accessible chromatin regions in iMGLs compared with phosphate-buffered saline (PBS)-treated iMGLs (Fig. 4a). HOMER^35^ was then used to identify transcription factor binding motifs enriched in the regions with increased accessibility, nominating transcription factors that may potentially regulate gene expression in response to ANs, including PU.1, MAFB, EGR1 and MITF (Supplementary Table 6).

**Extended Data Fig. 8:**
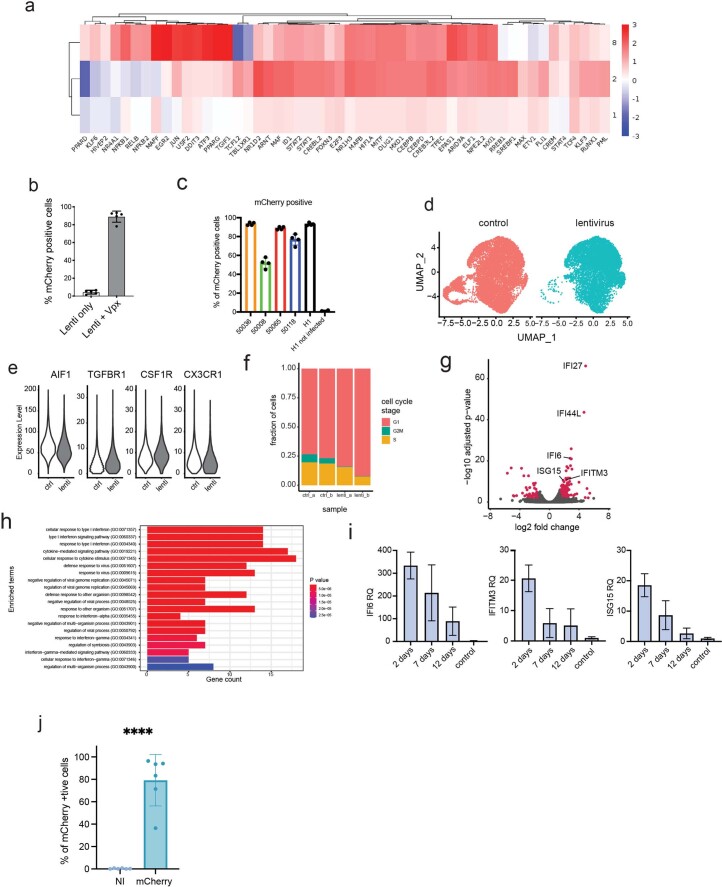
Lentivirus-mediated genetic modification of iMGLs. a) Heatmap of TFs identified by SCENIC DAM iMGL clusters (iMGL_1, iMGL_2 and iMGL_8), plotted by a scaled and centered area under the curve (AUC) for each regulon b) % of mCherry positive cells in iMGLs transduced with lentivirus only and lentivirus with VPX virus-like particles, determined by flow cytometry (p-value = 0.0079, two-tailed Mann-Whitney test). c) Transduction efficiency of iMGLs transduced from multiple cell lines using co-transduction strategy with VPX. Vector is non-targeting Cas9 control co-expressing mCherry and positive cells determined by flow cytometry d) UMAP projection of single-cell sequencing data from two untreated and lentivirus transduced iMGL differentiations, cells colored by treatment. e) Expression of AIF1, TGFBR1, CSF1R and CX3CR1 in the control and lentivirus samples. (AIF1 p-value = 0.463, TGFBR1 p-value = 0.999, CSF1R p-value = 0.999 and CX3CR1 p-value = 0.2193, Wald test with multiple hypothesis correction was performed with DESeq2). f) Distribution of cells by cell cycle stage in the control and lentivirus transduced samples. g) Volcano plot of differentially expressed genes between control and lentivirus transduced samples. Genes highlighted in red have adjusted p-value < 0.01 and log2 fold change < 1.5. Cycling cells were excluded prior to analysis. h) Gene ontology analysis of differentially expressed genes between control and lentivirus transduced samples. i) rtPCR time-course analysis of expression of three interferon-induced genes in iMGLs after lentivirus transduction. j) Percentage of mCherry-positive cells determined by flow cytometry, the number of cells transduced successfully (**** = p-value < 0.0001) (NI = non-infected). Error bars represent standard deviation.

To further identify regulators of microglial states, we performed a gene regulatory network analysis on iMGL single-cell profiles using SCENIC^36^. This identified the differential expression of transcription factors and their putative direct targets (together termed regulons) (Extended Data Fig. 8a and Supplementary Table 7). Analysis of the enriched regulons in clusters iMGL_1, iMGL_2 and iMGL_8 indicated that whereas some were shared across these three clusters (CEBPD, TCF4), several were differentially expressed in each cluster (ATF3 regulon in iMGL_8, REB1 in iMGL_2) (Extended Data Fig. 8a). Although we focused on DAMs, this analysis revealed potential regulatory networks underlying all iMGL clusters (Supplementary Table 7). Combining the transcription factors nominated by both chromatin accessibility and SCENIC analyses, we identified 16 putative regulators of the DAM state, including MITF, MAFB and EGR2 (Fig. 4b and Supplementary Table 6). We chose to focus on MITF as expression of this gene is upregulated in microglia of patients with AD^23,24^, and this transcription factor has been proposed as a regulator of the neurodegenerative signature^37^. Correspondingly, MITF mRNA is highly upregulated in DAM clusters iMGL_2 and iMGL_8 (Fig. 4c), and *MITF* expression scaled with the disease-associated trajectory identified in iMGLs (Extended Data Fig. 4a).

To functionally assess MITF in iMGL, we developed a lentiviral approach to transduce differentiated microglia, which are resistant to DNA delivery^19^. Transduction of monocytes with lentiviruses is facilitated by the SIV-encoded protein Vpx^38,39^, which degrades SAMHD1, a restriction factor that prevents reverse transcription of lentiviral RNA^40,41^. Co-delivery of Vpx packaged in virus-like particles (VLPs) led to ~89% transduction in iMGLs, compared with ~4% of iMGLs with lentivirus alone (Extended Data Fig. 8b). Vpx VLPs improved transduction of iMGLs derived from five stem cell lines (Extended Data Fig. 8c), indicating that Vpx has a robust effect regardless of the iMGL source. We performed scRNAseq and found that no-lentivirus (control) iMGLs and Vpx^+^ lentivirus-exposed iMGLs clustered together (Extended Data Fig. 8d) and had similar expression of key microglia markers (Extended Data Fig. 8e); however, fewer lentivirus-transduced iMGLs were proliferating (11.9%) compared with no-lentivirus iMGLs (24.8%) (Extended Data Fig. 8f). Although lentiviral transduction significantly increased the expression of 156 interferon-stimulated genes (Supplementary Table 8), assessment of a representative subset (*IFI6*, *IFITM3*, *ISGM3*) showed downregulation of these genes over 7 and 12 days compared with 2 days after transduction (Extended Data Fig. 8g–i). Thus, this transduction method is an efficient tool to manipulate gene expression in iMGLs while minimally affecting their transcriptional profile.

To understand MITF function, we transduced iMGL with a lentiviral vector expressing either MITF or mCherry (as a control) and performed gene expression and functional assays. On average, 79% of control lentivirus-transduced iMGLs were mCherry^+^ (Extended Data Fig. 8j). Bulk RNA-seq of iMGLs transduced with either MITF^42^ or mCherry (as control) indicated that MITF induced the differential expression of 670 genes compared with mCherry; of these, 300 genes were upregulated and 396 genes were downregulated (Fig. 4d and Supplementary Table 9), including *MITF*, confirming overexpression (Fig. 4e). Gene ontology analysis showed enrichment of cytokine signaling and cholesterol metabolism, pathways upregulated in the DAM transcriptional signature^6,23,24,34^, in MITF-transduced iMGLs compared with mCherry^+^ iMGLs (Fig. 4f). We observed a significant overlap between 38 genes upregulated in DAM-like iMGL_2 and iMGL_8 and MITF-transduced iMGLs (Fig. 4g and Supplementary Table 9), including *GPNMB* and *LPL* (Fig. 4i). Comparison of the overlap between the MITF-induced genes with iMGL markers of homeostatic, DAM, interferon-responsive, proliferative and antigen-presenting states highlighted a stronger overlap (as determined by hypergeometric test and number of genes) between MITF-induced genes and the DAM state compared to other microglia states (Fig. 4h). An increase in phagocytosis has been described in neurodegenerative disease models^4^, and we investigated the functional impact of MITF overexpression. MITF-transduced iMGLs exhibited increased phagocytosis of pHrodo-labeled ANs compared with mCherry^+^ iMGLs (Fig. 4j), suggesting that MITF regulates a phagocytic state in microglia. These observations identify MITF as a transcription factor that potentially regulates the neurodegenerative and phagocytic transcriptional signature in DAMs.

### Microglial state diversity is observed across multiple iPSC lines

**Fig. 5:**
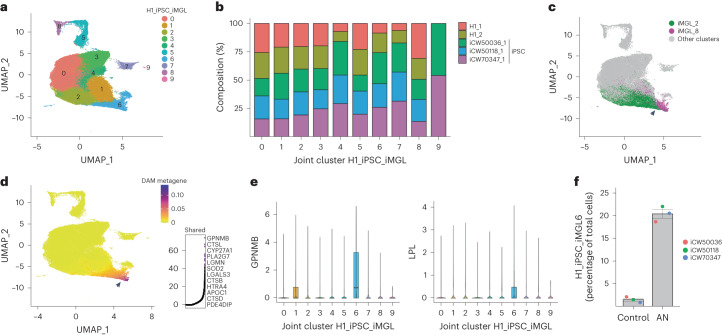
Multiple iPSC-line-derived iMGLs also exhibit transcriptional diversity and DAM induction. **a**, UMAP projection of integrated H1 iMGL and iPSC iMGL profiles; cells are colored by identity of H1_iPSC_iMGL joint clusters. **b**, Proportion of cells per H1_iPSC_iMGL joint cluster for each dataset (either iMGL H1 replicates or three separate iPSC lines differentiated into iMGLs). **c**, UMAP projection as in **a**. Green, iMGL_2; magenta, iMGL_8. **d**, UMAP projection (as in **a**) of the shared metagene common to both datasets in cluster 6. Right, top constituent genes of this shared factor. **e**, Violin plots showing expression of GPNMB and LPL across joint clusters in **a**. Boxes show the first and third quartiles of the data, with a line marking the median. Whiskers mark values closest to 1.5 times the interquartile range; no outliers are plotted. **f**, Percentages of nontreated (control) and AN-treated cells in joint cluster 6, from iPSC-derived iMGL only (two-tailed *t*-test, *P* = 0.00471; *n* = 3; mean and s.e.m. are shown). In **c** and **d**, the black arrow highlights joint cluster 6.

**Extended Data Fig. 9:**
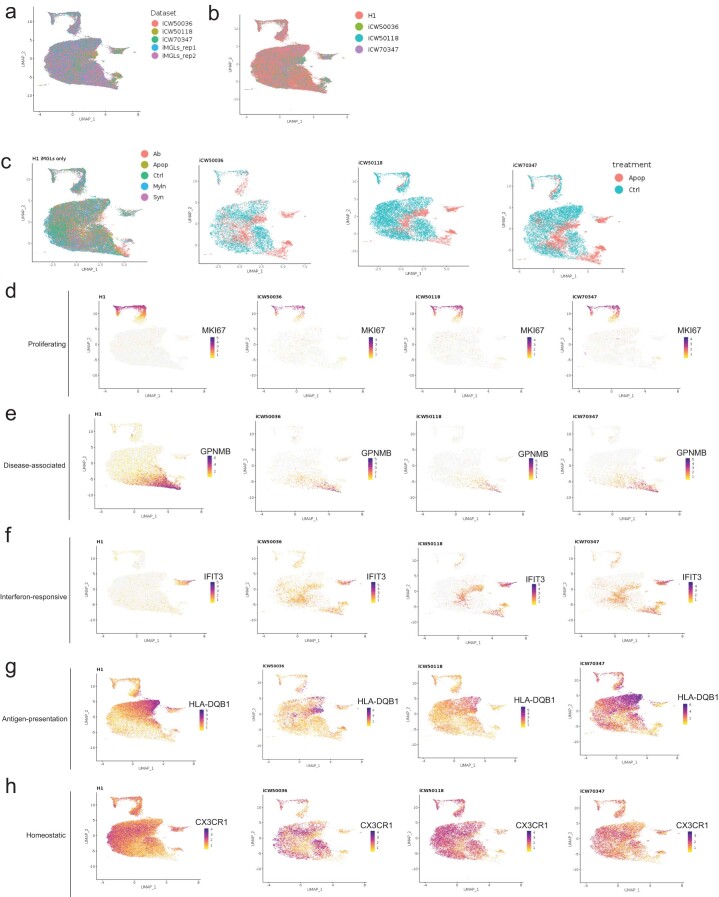
Comparison of H1 and iPSC-derived iMGLs revealed similar transcriptional signatures. a) UMAP projection of LIGER dataset integration of iMGL dataset and iPSC-derived iMGLs. Cells colored by dataset. b) UMAP projection (as in a), cells colored by line. c) UMAP projection (as in a), cells separated by line identity and colored by treatment condition. d-h) UMAP projection (as in a), cells separated by line identity and colored by expression of *MKI67 (d)*, *GPNMB (e), IFIT3 (f), HLA-DQB1 (g)* or *CXCR3 (h)*.

**Extended Data Fig. 10:**
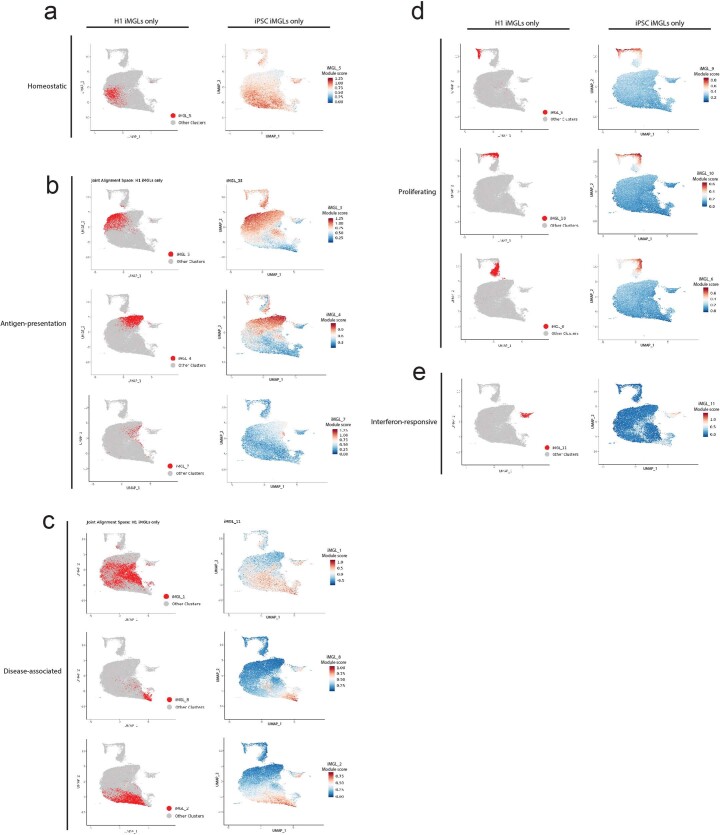
H1-derived and iPSC-derived iMGLs share similar transcriptional states, as demonstrated by module scores. UMAP projection of LIGER dataset integration of iMGL dataset and iPSC-derived iMGLs. Cells colored by iMGL cluster identity (left) or expression of corresponding module score (right) for homeostatic (a), antigen-presentation (b), disease-associated (c) proliferating (d), interferon-responsive (e) states.

A key advantage of stem-cell-derived models is the ability to perform parallel experiments on iPSC lines derived from multiple patients. To test whether our findings were broadly applicable, we differentiated iMGLs from three independent iPSC lines (iCW50118, iCW500036 and iCW70437) derived from healthy individuals with APOE 3/3 status (as previously defined^43^) and no *TREM2* mutations. We exposed these iMGL ANs or PBS, and performed scRNA-seq. This analysis generated 41,655 iPSC-derived single-cell transcriptomes, which we integrated with our iMGL dataset (here referred to as the H1 dataset) using LIGER (Fig. 5a,b and Supplementary Table 10). We found a high level of similarity between the two datasets (alignment score, 0.973; Fig. 5b and Extended Data Fig. 9a–c). This integrative analysis produced ten joint clusters (H1_iPSC_iMGL0-9). Most joint clusters were well distributed between H1-derived and iPSC-derived iMGLs, with the exception of H1_iPSC_iMGL9; this was only present in iMGLs from two iPSC lines (Fig. 5b), which contained rare (100 of 41,655) cells enriched for ribosomal genes (for instance, *RPS18*). Further analysis indicated similar transcriptional states to those identified previously as determined by marker gene expression (Extended Data Fig. 9d–h) and module score calculation (Extended Data Fig. 10). Based on this analysis, DAM clusters iMGL_2 and iMGL_8 mapped to H1_iPSC_iMGL_6 (Fig. 5a,c,d), which corresponded to a metagene consisting of DAM signature genes (Fig. 5d and Extended Data Fig. 10c) including canonical marker genes *GPNMB* and *LPL* (Fig. 5e). We found that iPSC-derived iMGLs from joint cluster H1_iPSC_iMGL_6 were significantly increased as a proportion of total iMGLs following exposure to ANs versus no treatment (Fig. 5f). This indicates that microglia differentiated from iPSCs also exhibit the DAM state in the presence of ANs and highlights the generalizable nature of the iMGL platform.

## Discussion

Here, we provide a toolbox for the study of human microglial states, including a single-cell gene expression resource for in vitro iMGL. We found that challenge with CNS substrates produced a large degree of transcriptional diversity and that the iMGL states induced in vitro mapped to those found in the human brain. Exposure to some CNS substrates was sufficient to induce a TREM2-dependent DAM state that was analogous to those found in both the human and mouse brain^6,20,44^, suggesting that addition of easily sourced substrates can produce models of human microglial states in vitro. We also demonstrated that these features are shared over multiple iPSC lines, which will enable investigators to analyze microglia from a full spectrum of patients and diseases, and prioritize human cell lines for in-depth functional characterization^45^.

One key finding was that iMGLs existed in distinct transcriptional states in vitro even in the absence of stimulation, and that upon stimulation with a specific substrate, these states did not converge to a single transcriptional signature. This was similar to in vivo observations that microglia in similar locations could adopt distinct transcriptional states^5^ and suggests that heterogeneous response to substrates is common in microglia. However, microglial profiling ex vivo represents only a snapshot of the transcriptional diversity at a particular time after treatment and may not reflect the entire dynamic process. While the dynamics and plasticity of microglial states after a stimulus remain unclear^14^; this in vitro platform will enable future longitudinal and imaging studies to address this important question.

An additional challenge is that microglia, unlike other brain cell types, are not amenable to viral transduction. The development of genetically modified iPSCs is low throughput and costly, and deletion of essential genes can affect differentiation^46^. To further enable scalable experiments in iMGLs, we developed a lentiviral protocol that broadly transduced iMGLs in the last phase of their differentiation, inducing only modest perturbation of the core microglial transcriptional signature. We applied this technology to interrogate the function of MITF, a transcription factor that we identified as a putative regulator of DAM, which is also upregulated in AD^23,24,37^. We found that MITF overexpression drives a subset of the DAM signature and increased phagocytosis. Although the number of overlapping genes between microglia with MITF overexpression and those in the DAM state was relatively modest, gene ontology analysis of all MITF-driven genes converged on known pathways upregulated in the DAM transcriptional signature across species^6,23,24,34^, suggesting that MITF is a potential regulator of the neurodegenerative-associated signature and function^23,24,37^. By allowing facile genetic modification of iMGLs, this technology has the potential to enable the identification of key regulators of microglial states and functions^14,27,47^ and the rapid application of molecular tools.

Although we focused on the DAM signature, the data provided here will enable the dissection of many other microglial states, such as interferon-responsive or proliferative microglia^14,27^. While our analyses suggested that iMGLs adopted states analogous to those found in vivo, we caution that this does not mean the signatures described here were identical. For example, although iMGLs and human brain biopsy DAMs shared many aspects of their gene signatures, there were also differences. Similarly, modeling baseline homeostatic states in vitro is notoriously challenging^15,17^, and further optimization will be needed to enhance the in vitro microglial homeostatic signature. We envision this resource as a tool for investigators to examine the function of human microglial diversity in a high-throughput manner. These studies could then be augmented and validated with lower-throughput approaches that recapitulate the in vivo environment, such as organoid or chimeric models^16,26,32,48,49^.

Combining substrate exposure, scRNA-seq, integrative analyses, epigenetics and viral vectors will allow manipulation of the genes that define microglial states and rapidly assay their impact on microglial functions. The use of additional substrates (for example, different forms or species of amyloid, tau and synuclein) may enable the modeling of microglial states found in other disorders. This platform also allows the generation of large numbers of cells for unbiased characterization methods such as proteomics or epigenetics, which are challenging in human tissue because of the small number of microglia that can be obtained from a single individual. Finally, combining iMGLs with neuron and/or astrocyte cocultures^50^ could enable the study of noncell-autonomous phenotypes and neuron–glia immune interactions. In conclusion, the in vitro approach described here will enable a broad characterization of human microglial states and bridge the gap between the transcriptomic profile and functional role of these states in neuroimmune interactions in health and disease.

## Methods

### Embryonic stem cell and iPSC lines

All stem cell work was reviewed and approved by the Broad Institute Office of Research Subject Protection. Unless otherwise stated, H1 embryonic stem cells (WiCell) were used. The iPSC lines were obtained from the California Institute of Regenerative Medicine (CIRM) hPSC Repository funded by CIRM: CW50118, CW50008, CW50065, CW500036 and CW70437. The cell lines chosen for scRNA-seq (CW50118, CW500036 and CW70437) were confirmed as APOE 3/3 status with no *TREM2* mutation by Sanger sequencing. In addition, individuals did not exhibit cognitive decline at the time of collection, which was after 70 years of age for all lines (Supplementary Table 10), suggesting that these lines should represent healthy individuals. The TREM2-knockout and isogenic control iPSC lines were as previously characterized^20^ and were obtained from M. Blurton-Jones. These lines were derived from cell line AICS-0036-006 from the NIGMS Human Genetic Cell Repository at the Coriell Institute for Medical Research; thus, they have not undergone the standard quality control of the Repository.

### iMGL differentiation

iMGLs were differentiated as previously described^21^. Briefly, iPSC or embryonic stem cells were cultured in Essential 8 (E8) (Thermo Fisher Scientific) media on six-well plates coated with Matrigel (Corning). When confluent, cells were dissociated using Accutase (Stem Cell Technologies), centrifuged for 5 min at 300*g* and counted using trypan blue (Thermo Fisher Scientific). Next, 200,000 cells per well were resuspended in E8 containing 10 μM Y-27632 ROCK inhibitor (Selleckchem) in a low-adherence six-well plate (Corning). For the first 10 days, cells were cultured in HPC medium (50% IMDM Thermo Fisher Scientific), 50% F12 (Thermo Fisher Scientific), ITSG-X 2% v/v (Thermo Fisher Scientific), l-ascorbic acid 2-phosphate (64 μg ml^−1^, Sigma), monothioglycerol (400 mM, Sigma), poly(vinyl) alcohol (10 mg ml^−1^, Sigma), GlutaMAX (1×, Thermo Fisher Scientific), chemically defined lipid concentrate (1×, Thermo Fisher Scientific) and nonessential amino acids (Thermo Fisher Scientific)). At day 0, embryoid bodies were gently collected, centrifuged at 100*g* and resuspended in HPC medium supplemented with 1 μM ROCK inhibitor, FGF2 (50 ng ml^−1^, Thermo Fisher Scientific), BMP4 (50 ng ml^−1^, Thermo Fisher Scientific), Activin-A (12.5 ng ml^−1^, Thermo Fisher Scientific) and LiCL (2 mM, Sigma), then incubated in a hypoxic incubator (5% O_2_, 5% CO_2_, 37 °C). On day 2, cells were gently collected, and the medium was changed to HPC medium supplemented with FGF2 (50 ng ml^−1^, Thermo Fisher Scientific) and VEGF (50 ng ml^−1^, PeproTech), before cells were returned to the hypoxic incubator. On day 4, cells were gently collected and the medium was changed to HPC medium supplemented with FGF2 (50 ng ml^−1^, Thermo Fisher Scientific), VEGF (50 ng ml^−1^, PeproTech), TPO (50 ng ml^−1^, PeproTech), SCF (10 ng ml^−1^, Thermo Fisher Scientific), IL6 (50 ng ml^−1^, PeproTech) and IL3 (10 ng ml^−1^, PeproTech); then, cells were incubated in a normoxic incubator (20% O_2_, 5% CO_2_, 37°C). On days 6 and 8, 1 ml of day 4 media was added to each well. On day 10, cells were collected, counted using trypan blue and frozen in Cryostor (Sigma Aldrich) in aliquots of 300,000–500,000 cells.

To start iMGL differentiation, cells were thawed, washed 1× with PBS and plated at 100,000–200,000 cells per well in a six-well plate coated with Matrigel in iMGL media ((DMEM/F12 (Thermo Fisher Scientific), ITSG (2% v/v, Thermo Fisher Scientific), B27 (2% v/v, Thermo Fisher Scientific), N2 (0.5% v/v, Thermo Fisher Scientific), monothioglycerol (200 mM, Sigma), GlutaMAX (1×, Thermo Fisher Scientific), nonessential amino acids (1×, Thermo Fisher Scientific)) supplemented with M-CSF (25 ng ml^−1^, PeproTech), IL-34 (100 ng ml^−1^, PeproTech) and TGFB-1 (50 ng ml^−1^, PeproTech). Cells were fed every 2 days and replated at day 22. On day 30, cells were collected and replated in iMGL media supplemented with M-CSF (25 ng ml^−1^, PeproTech), IL-34 (100 ng ml^−1^, PeproTech), TGFB-1 (50 ng ml^−1^, PeproTech), CD200 (100 ng ml^−1^, VWR) and CX3CL1 (100 ng ml^−1^, PeproTech) to a final concentration of 40,000 cells cm^−2^. Cells were used at day 40 for functional and transcriptomic assays. iMGL differentiation was assessed at day 10 (expression of CD43 and CD45) and day 40 (expression of CD45, CD11B, P2RY12, CX3CR1 and DAPI for viability) by flow cytometry (see below for protocol). During the quality control on day 40, we observed similar protein expression among the replicates, with the exception of CX3CR1, which had lower expression in replicate 1 (Extended Data Fig. 1a). At no point were iMGLs exposed to serum of any kind. Throughout the differentiation, cell viability was >90% as measured by trypan blue.

### Flow cytometry

For sample processing, iMGLs were detached using cold PBS then resuspended in fluorescence-activated cell sorting (FACS) buffer (PBS containing 2% bovine serum albumin and 0.05 mM EDTA). Samples were incubated for 15 min in human Fc block (BD Biosciences), followed by 1 h staining with conjugated antibodies (see below) at 4 °C. Samples were washed three times with FACS buffer and resuspended in 500 μl of FACS buffer for flow cytometry. Samples were run on a CytoFLEX S analyzer (Beckman Coulter) until at least 2,000 cells have been recorded. For analysis, cells were identified according to the following gating: (1) cell versus debris (FSC-A versus SSC-A); (2) singlets (FSC-A versus FSC-H), and antibody-specific gating based on a negative control sample. Antibodies for staining iMGLs were CD45-FITC (BioLegend), CD11B-APC-750 (BioLegend), P2RY12-PB450 (BioLegend), Cx3CR1-PrCp (BioLegend) 1/500 for all.

For phagocytosis, cells were treated with substrates labeled with pHrodo Green (see below) for 24 h. iMGLs were then washed with PBS and collected in FACS buffer (PBS containing 2% bovine serum albumin and 0.05 mM EDTA). Samples were then directly run on a CytoFLEX S analyzer (Beckman Coulter) until at least 2,000 cells had been recorded. For analysis, cells were identified according to the following gating: (1) cell versus debris (FSC-A versus SSC-A); (2) singlets (FSC-A versus FSC-H); (3) viability (based on DAPI stain). Phagocytosis was quantified as the mean fluorescence intensity of pHrodo for each sample. Each experiment was performed using multiple independent differentiations, and each dot in the corresponding graph represents a biological replicate.

All analyses were performed using FlowJo v.9, with Prism9 for statistical analysis.

### CNS substrate isolation and iMGL treatment

Unless otherwise stated, iMGLs were treated with CNS substrates for 24 h before transcriptomic, flow cytometry or RNAscope analysis.

Synaptosomes were prepared from rodent brains as described previously^51^. Briefly, C57BL/6J mice were euthanized with CO_2_; then, brains were dissected and homogenized in HEPES-buffered sucrose (0.32 M, 5 mM HEPES, pH 7.4). The resulting homogenate was spun at 800–1,200*g* to separate the nuclear fraction. A further spin at 15,000*g* was carried out to generate crude synaptosomes, and 3.1 μg cm^−2^ was used for experiments.

Myelin was isolated from C57BL/6J mice. Animals were transcardially perfused with cold HBSS, and whole brains were manually homogenized in RPMI. Samples were applied to a Percoll gradient and, after a 30-min spin at 500g, the top layer was collected. Myelin was washed twice with water, and 3.1 μg cm^−2^ was used for experiments.

ANs were generated using either SH-SY5Y cells or iNeurons generated as previously described^44^, using neurogenin-2 (NGN2) for 14 days. Briefly, stem cells were grown in StemFlex (Stem Cell Technologies) and grown on plates coated with Matrigel (Corning) plates at 37 °C and 5% CO_2_. Cells were infected with TetO-Ngn2-Puro and with rtTA lentiviral particles in StemFlex medium with 1 µM ROCK inhibitor Y-27632 for 24 h; they were then passaged, and differentiation was started when cells reached 70–80% confluence. For the first 2 days, cells were grown in N2 media (DMEM/F12 (Life Technologies, 11320-033), N2 supplement (0.5% v/v, Gibco), 1× GlutaMAX (Gibco), 0.1 mM nonessential amino acids (Gibco), 0.5% glucose, doxycycline hyclate (2 µg ml^−1^)). On day 0, N2 media was supplemented with 0.1 µM LDN, 5 µM XAV and 10 µM SB; and on day 2, the cells were fed with N2 media with no supplement. On day 3, cells were replated to 100,000 cells per well in a six-well plate and transferred to neurobasal media (Neurobasal (Gibco), 1× B27 (Gibco), 1× GlutaMAX (Gibco), 0.1 mM nonessential amino acid (Gibco), 0.5% glucose, doxycycline hyclate (2 µg ml^−1^)) supplemented with 10 ng ml^−1^ CNTF, 10 ng ml^−1^ BDNF and 10 ng ml^−1^ GDNF. Cells were fed every other day until day 14. Mature cells were submitted to ultraviolet radiation (500 J m^−2^ using an ultraviolet cross-linker), gently collected 24 h later, washed in PBS and counted. On day 40, 35,000 ANs per cm^2^ were added to the iMGL for each experiment.

For conjugation to pHrodo (Red SE or Green, STP Ester; Thermo Fisher Scientific), labeling was done according to the manufacturer’s protocol. Briefly, substrates were incubated with 1 ml pHrodo per 1 mg substrate for 2 h at room temperature, protected from light, washed ten times using PBS and frozen in 5% dimethyl sulfoxide. Only synaptosomes, myelin or ANs were conjugated.

Amyloid fibrils were prepared from fluorescently labeled Aβ (Beta-Amyloid (1-42) HiLyte Fluo, AS-60479-01, Anaspec). Aβ peptides corresponding to amino acids 1–42 were dissolved in sterile water to 100 μg ml^−1^ concentration, vortexed thoroughly and shaken in a 37 °C incubator for 4 h. Then, the Aβ peptides were incubated for 5 days at 37 °C to fibrilize. Amyloid fibrils were then mixed and delivered to cells at 5 μg ml^−1^. Fresh amyloid fibrils were created for each experiment.

Phagocytosis of substrates was tested by flow cytometry. Although we observed variability in phagocytosis for amyloid fibrils among replicates, all other substrates were readily and consistently phagocytosed (Extended Data Fig. 3b).

### Sample preparation for scRNA-seq

For single-cell sequencing experiments, iMGLs at day 40 were treated with myelin, synaptosomes, ANs or amyloid fibrils for 24 h (see above). Cells were washed with warm media to remove floating cells and detached in PBS on ice. Cells were then centrifuged and resuspended in cold PBS at 1,000 cells per µl.

For the single-cell sequencing experiment comparing control versus lentivirus-transduced cells, iMGLs were transduced with a nontargeting (NT) guide RNA–Cas9–mCherry lentivirus (see below for more details on lentiviral vector, production and exposure) on day 35. Cells were exposed to the virus overnight, and 100% of the medium was changed the next morning. Cells were then cultured for 7 more days and fed on a regular schedule. On day 7, mCherry expression was confirmed, and all media were removed. Cells were washed with warm media to remove floating cells and detached in PBS on ice. Cells were then centrifuged and resuspended in PBS.

After resuspension and counting, cells were loaded into the 10x Chromium V3 system (10x Genomics). Reverse transcription and library generation were performed according to the manufacturer’s protocol. Owing to a counting error, the number of cells loaded for replicate 1 of the H1 scRNA-seq dataset was approximately threefold lower than that for replicate 2, necessitating a data integration strategy for batch correction (see below). Sequencing was performed on a NovaSeq S2 (Illumina).

### Quantitative real-time PCR

iMGLs on day 40 were treated for 24 h and collected using RLT buffer. RNA extraction was done using an RNease Plus mini kit (Qiagen) according to the manufacturer’s protocol. For quantitative rtPCR, a TaqMan RNA-to-Ct 1-step kit (Thermo Fisher Scientific) was used according to the manufacturer’s protocol using the following TaqMan probes (Thermo Fisher Scientific): GAPDH (HS02786624_G1) and GPNMB (HS01095669_M1). Quantification was done using the 2 ^−^^ΔΔCT^ method^52^. Each biological sample was measured in triplicate, and the average for each biological sample is shown in the corresponding graph. Statistics were analyzed using Prism9 software, and graphs show individual cells with mean and s.d. Two-tailed *t*-tests were used to determine significance.

### Immunocytochemistry, imaging and quantification

iMGLs plated on Matrigel-coated coverslips were fixed in 4% paraformaldehyde (PFA) for 15 min, followed by permeabilization (0.2% Triton-X in PBS) for 10 min and blocking (5% normal donkey serum in 0.025% Triton-X/PBS) for 30 min. iMGLs were incubated with primary antibodies overnight (concentrations below) and washed three times for 10 mins with 0.025% Triton-X/PBS. Then, cells were incubated with a secondary antibody (Thermo Fisher Scientific) for 60 min at room temperature. Following three washes for 10 min each with 0.025% Triton-X/PBS, coverslips were mounted on slides using ProLong Gold antifade (Thermo Fisher Scientific).

The primary antibodies used and their concentrations were as follows: TREM2 (R&D systems, AF1828-SP) 1/100, GPNMB (Cell Signaling, E1YZJ) 1/500, APOE clone 6B9 (Helmholtz Antibody Collection) (1/100). More information on the APOE antibody can be found at https://www.alzforum.org/alzantibodies/apoe-clone-6b9. The secondary antibodies used and their concentrations were: donkey anti-rabbit 488 (Thermo Fisher Scientific) 1/500, goat anti-rabbit 594 (Thermo Fisher Scientific) 1/500.

Imaging was performed on an Andor CSU-X spinning disk confocal system coupled to a Nikon Eclipse Ti microscope equipped with an Andor iKon-M camera. Images were acquired using a ×60 oil objective (Nikon). All images shown are representative images taken from at least three independent experiments. Statistics were analyzed using Prism9 software, and graphs show individual cells with mean and s.d. Two-tailed *t*-tests were used to determine significance.

### In situ hybridization RNAscope and quantification

RNAscope (Advanced Cell Diagnostics) was carried out according to the manufacturer’s protocol for cultured adherent cells on coverslips. Briefly, iMGLs plated on Matrigel-coated coverslips were fixed for 30 min using 4% PFA and dehydrated (5 min 50% EtOH, 5 min 70% EtOH, 2× 5 min 100% EtOH) and kept at −20 °C until use. When ready for treatment, cells were subjected to the RNAscope Multiplex Fluorescent V2 Assay (Advanced Cell Diagnostics), and probes against human APOE (catalog no. 433091), ABCA1 (catalog no. 432291) and C1QA (catalog no. 485451-C2) were used at the recommended concentrations. TSA Plus fluorophores (Perkin Elmer) were used at 1:1500 concentration.

Imaging was performed on an Andor CSU-X spinning disk confocal system coupled to a Nikon Eclipse Ti microscope equipped with an Andor iKon-M camera. Images were acquired using a ×60 oil objective (Nikon). C1QA was used to identify regions of interest to identify individual cells. As microglia change shape and can become ameboid in response to stimuli, quantification of intensity could not be normalized to the cell’s area; therefore, only the fluorescence intensity of the target probe was measured using Fiji^53^. Statistics were analyzed using Prism9 software, and graphs show individual cells with mean and s.d. Two-tailed *t*-tests were used to determine significance.

### Production of lentivirus and Vpx VLPs

Lentivirus and Vpx VLPs were produced by transfecting HEK293T cells using TransIT-LT1 reagent (Mirus). For lentivirus, the following plasmids were transfected (amounts per well for a six-well plate): 1.6 μg mCherry-expressing and Cas9-expressing lentiviral genome plasmid pXPR_BRD044 (obtained from the Broad Institute Genome Perturbation Platform), 0.4 μg pCMV-VSV-G (Addgene no. 8454) and 1 μg psPAX2 (Addgene no. 12260). For Vpx VLPs, 0.4 μg pCMV-VSV-G and 2.6 μg pSIV3 Vpx plasmids were transfected. We achieved identical results with both an in-house Vpx plasmid (pSIV3) and pSIV-D3psi/delta env/delta Vif/delta Vpr^54^ (Addgene no. 132928). Media were changed to OptiMEM 18 h after transfection (2 ml per well in a six-well plate). Two days after transfection, viral supernatant was harvested and centrifuged at 500*g* for 10 min to remove any cells in the supernatant. Clarified supernatant was concentrated 10× by incubation with Lenti-X Concentrator (Takara) overnight (owing to the size of the viral genome, the virus had to be concentrated to achieve sufficient titer). The mixture was spun down the next day per the manufacturer’s instructions and resuspended in base iMGL media. Vpx VLPs were not concentrated. Lentivirus was titered using Lenti-X p24 Rapid Titer Kit (Takara). Both lentivirus and Vpx VLPs were flash frozen, stored at −80 °C and thawed on ice before use.

### Lentivirus transduction for FACS, single-cell sequencing and quantitative rtPCR time-course study of the effects of lentiviral transduction of iMGLs

For FACS, iMGLs were seeded in a 96-well plate. To transduce iMGLs, we added 53 ng of p24 per 10,000 cells of lentivirus and 10 μl per 10,000 cells of Vpx. Lentivirus and Vpx VLPs were added on day 35. The next day, all media were removed and replaced with fresh media, which were then changed every 2 days. On day 42, cells were detached as described above and FACS was performed to quantify mCherry expression from lentivirus.

For single-cell sequencing, 200,000 iMGLs were seeded in a six-well plate, where one well corresponded to one replicate for single-cell sequencing, and treated with lentivirus and Vpx VLPs as described above. Controls were treated the same as lentivirus in terms of media changes. Seven days after virus addition, cells were detached with cold PBS on ice for 10 min and spun at 300*g* for 5 min. Cells were then resuspended in a small volume of PBS and counted. Approximately 20,000 cells were used as input for each single-cell sequencing replicate.

For the time-course study of expression of interferon-stimulated genes after exposure to lentivirus, iMGLs were seeded into 24-well plates. On day 35, cells were cotransduced with lentivirus and Vpx VLPs. RNA was harvested on days 37, 42 or 42. To harvest RNA, iMGL media were removed and RLT Plus buffer from an RNeasy kit (Qiagen) was added directly to the wells. RNA was purified following the manufacturer’s instructions. For quantitative rtPCR, a TaqMan RNA-to-Ct 1-step kit (Thermo Fisher Scientific) was used according to the manufacturer’s protocol using the following TaqMan probes (Thermo Fisher Scientific): GAPDH (HS02786624_G1), IFI6 (Hs00242571_m1), IFITM3 (Hs03057129_s1) and ISG15 (Hs01921425_s1). Data were analyzed using CFX Maestro software (BioRad). For all quantitative rtPCR experiments, biological replicates are shown. Error bars represent s.d.

### ATAC-seq library preparation and sequencing

ATAC-seq libraries were prepared essentially as described^55^, with the following modifications. For each sample, 10,000 viable frozen cells were used per library construction. We optimized the number of PCR cycles by stopping PCR after five cycles, taking an aliquot from the partially amplified library, and performing quantitative PCR for 25 cycles. We visually examined the quantitative PCR amplification profile to determine the number of cycles required to reach one-third of the plateau and extended the original PCR by this number of additional cycles. Libraries were sequenced on an Illumina NextSeq platform using a 75-cycle NextSeq 500 high-output V2 kit (read 1: 36 cycles; index 1: eight cycles; index 2: eight cycles; read 2: 36 cycles).

### MITF-overexpression lentivirus

For MITF overexpression, lentiviral vectors contained either MITF (NM_198159.3) or mCherry under the EF1α promoter^42^. Lentivirus was produced as described above. At day 35, iMGLs were infected with MITF-expressing or mCherry-expressing lentivirus, and the media were changed the following day. At day 42, iMGLs were treated for functional or transcriptomic characterization.

### Bulk RNA-seq library preparation and sequencing

iMGLs were collected using RLT buffer (Qiagen), and RNA extraction was done using an RNeasy Plus mini kit (Qiagen) according to the manufacturer’s protocol. Quantification was performed and RNA integrity was assessed using an RNA 600 Pico chip on a Bioanalyzer (Agilent). RNA (5 ng) was used as input for library constructions. Libraries were constructed using a NEBNext Poly(A) mRNA magnetic isolation module (NEB E7490) in combination with NEBNext Ultra RNA library prep for Illumina (NEB E7530) following the manufacturer’s instructions. Libraries were quantified using an Agilent high-sensitivity DNA chip and sequenced on NextSeq (Illumina). Samples were sequenced in four flow chambers, and reads from each chamber were merged to generate a final fastq final for each sample.

### scRNA-seq preprocessing, quality control and general analysis

Cell Ranger (v.3.1, 10x Genomics) was used for demultiplexing, barcode preprocessing, generation of fastq files, alignment (to GRCh38-2020-A/GENCODE v32/Ensembl 98) and counting of unique molecular identifiers (UMIs). Owing to differences in sequencing depth between replicates (see above), we applied Cell Ranger’s aggr function to all samples to merge and normalize by sequencing depth.

All downstream analyses (see below for specific analyses) were performed in R using the LIGER (v.0.5) and Seurat (v.2.3.4 or v.3.2.1) packages^30,56^. Lentivirus-exposed iMGLs were excluded from this analysis, and analysis of this experiment is described below. All data and metadata (replicate and condition) were incorporated into a single object, and the percentage of mitochondrial RNA per cell was calculated. We only considered cells with the following characteristics: (1) number of genes: 2,000–7,000; (3) number of UMIs: 500–6,000; (3) percentage mitochondrial RNA 0–0.2%. This yielded 57,837 single iMGL cells.

Cells of interest were subsetted and converted to a LIGER object for integration. To integrate the two replicates, we considered the replicate (1 or 2) as the LIGER dataset variable and applied the LIGER pipeline for dataset integration (*k* = 20, lambda = 5). Clustering was performed using the louvainCluster function (clustering resolution = 0.7), followed by merging of clusters with fewer than 45 unique differentially expressed genes. Clusters were merged based on hierarchical clustering of iMGL clusters by differential gene expression. A small population of cells activated by manual handling (ExAM signature, see below for score calculation, 1116 cells) were removed from the final dataset. In addition, another small set of cells of unknown identity, which had low microglial identity scores, were removed (267 cells). These two filtration steps removed a total of 1383 (2.4%) of the 57,837 cells, resulting in a final dataset of 56,454 (Fig. 1). A UMAP embedding was calculated using LIGER’s runUMAP function. The alignment was evaluated with the CalcAlignment and CalcAgreement LIGER functions. All gene expression, metagene and violin plots were produced using LIGER functions and modified using ggplot2. Cell cycle scoring was performed using the CellCycleScoring function in Seurat.

### Module score for microglia identity, maturity and artifactual activation of microglia (ExAMs)

The module scores for testing for microglial identity and maturity were created using published gene lists^15,21^. For human microglial identity^21^, the following genes were used: P2RY12, *GPR34*, *C1QA*, *CX3CR1*, *CABLES1*, *BHLHE41*, *TREM2*, *OLFML3*, *PROS1*, *APOE*, *SLCO2B1*, *SLC7A8*, *PPARD* and *CRYBB1*. For microglial maturity, the top 30 enriched genes identified in mature human microglia^15^ were used: *SPP1*, *CD74*, *ACTB*, *C3*, *FTL*, *FOS*, *CSF1R*, *B2M*, *C1QC*, *C1QB*, *PSAP*, *A2M*, *ITM2B*, *LAPTM5*, *CTSB*, P2RY12, *C1QA*, *SLCO2B1*, *RGS1*, *APOE*, *CCL4L2*, *RNASET2*, *NEAT1*, *CX3CR1*, *DUSP1*, *SAT1*, *ZFP36*, *CD81*, *HLA-B* and *HLA-DRA*. The module score for testing for artifactual gene expression was created using genes previously identified as being upregulated during single-cell isolation and cell handling^22^. These genes were: *RGS1*, *HIST2H2AA1*, *HIST1H4I*, *NFKBIZ*, *KLF2*, *JUNB*, *DUSP1*, *CCL3*, *HSPA1A*, *HSP90AA1*, *FOS, HSPA1B*, *JUN*, *JUND*, *NFKBID, GEM*, *CCL4*, *IER5*, *TXNIP*, *HIST1H2BC*, *ZFP36*, *HIST1H1C*, *EGR1*, *ATF3* and *RHOB*. In all cases, the module score was implemented in Seurat using the AddModuleScore function with a control size of 25.

### Module score for cluster identity

The module score for cluster identity was calculated using only positively enriched genes from a published xenotransplanted iMGL single-cell study^26^. The module score was implemented in Seurat using the AddModuleScore function with a control size of 100. Larger and smaller control sizes were tested with no observed impact on results (data not shown).

### Single-cell differential expression analysis and UpSet plots

To identify differentially expressed genes per LIGER cluster, we used the MAST package (v.1.12.0)^57^ and included the number of UMIs and number of genes as covariates. Only genes expressed in more than 10% of cells per cluster were considered. Cell gene expression was then scaled, centered and averaged per cluster before heatmap generation. Heatmaps were generated using the ComplexHeatmap R package. To calculating the number of unique differentially expressed genes, UpSet plots were created for each state using all differentially expressed genes with the UpSetR R package.

### Monocle trajectory analysis

For trajectory analysis of AN-exposed iMGLs, normalized gene expression data were aligned by replicate using Batchelor^58^, which was chosen owing to compatibility with the Monocle3 pipeline. Dataset integration across replicates with Batchelor gave similar results to those obtained with LIGER and Seurat (data not shown). The preprocessing, dimensionality reduction and pseudotime ordering of cells were performed using Monocle3 (ref. ^28^), and cluster labels from the initial LIGER analysis (see above) were used for analysis and plotting.

### Cluster proportions, differential abundance test and fold change analysis

For plotting cell proportions, datasets were downsampled to the same size randomly using the groupdata2 or LIGER R packages. The percentage contribution of each condition to a cluster by replicate was then calculated and averaged. To determine statistical significance of cluster abundances across conditions, we used counts (not proportions) and implemented Dirichlet regression, a multivariate test that accounts for overall composition per sample, using the DirichletReg R package (https://github.com/maiermarco/DirichletReg), v.0.7, and a previously published workflow^59^. Fold change values were calculated by replicate, relative to the control condition, and a log_2_ transformation for final log fold-change plots was reported.

### GSEA of iMGL clusters and other datasets

GSEA was run using the fgsea package^60^ (v.1.12.0). Genes for each iMGL cluster were ordered by log(fold change). Pathways were defined as positively differentially enriched genes identified by differential expression analysis in other studies: Hasselmann et al.^26^, Gerrits et al.^24^ and the meta-analysis described in Gazestani et al.^29^. Data were plotted using the ComplexHeatmap R package^61^.

### LIGER integration of iMGLs with different datasets

For large dataset integrations (that is, those with large numbers of cells, individuals or both) we used LIGER because of both its scalability and its performance on large datasets. For analysis of the human cortical biopsy microglia, the final microglia object, cluster labels and differentially expressed genes from the Gazestani et al. object were used. Nonmicroglial cells, including myeloid and peripheral macrophages, were excluded before analysis. For analysis of the mouse DAM dataset, the data were reprocessed and analyzed using Seurat (v.3.2.1), and cluster identities (in particular, the identification of DAM microglia) were determined based on differential gene expression and comparison with the source paper (for instance, upregulation of *Lpl*, *Clec7a*, *Trem2*, *Itgax*, *Cd9* and *Axl*). For xMG analysis, the cluster assignments from the original paper were used.

All cross-context (xenotransplanted iMGLs) and cross-species (human in vivo and mouse in vivo) dataset integrations were performed using the LIGER package^30^. Post-QC (quality control) iMGL count data were used for all alignments. For each alignment, the optimal *k* and lambda values were identified using the appropriate LIGER functions. For each alignment, the data, parameters and dataset variable arrangement were as follows.

**Table:** 

Data	Reference	Parameters	Dataset variable
Human cortical brain biopsy microglia	Gazestani et al.^29^	*k* = 20, lambda = 10, clustering resolution = 0.3	iMGL replicates (untreated and all CNS substrates), individual patients (51 datasets)
Mouse in vivo: DAMs	Keren-Shaul et al.^6^	*k* = 20, lambda = 5	iMGL replicates, mouse microglia as one dataset
Xenotransplanted iMGLs: xMGs	Hasselmann et al.^26^	*k* = 20, lambda = 5	iMGL replicates, xMG in WT, xMG in 5xFAD

Note: LIGER alignment of H1 iMGLs with iPSC-derived iMGLs (Fig. 5) is described below. For the human cortical brain biopsy dataset integration, two small clusters of doublets (with marker genes for other brain cell types) were identified and removed before downstream analysis.

These alignments were evaluated with the CalcAlignment and CalcAgreement LIGER functions. All gene expression plots and violin plots were produced using LIGER functions and modified using ggplot2.

### Hypergeometric test comparing human and iMGL DAM gene expression

Before performing the hypergeometric test, both iMGL and in vivo human DAM genes were identified. DAM genes in iMGLs were determined by taking the union of differentially expressed genes for iMGL_02 and iMGL_8 (see Single-cell differential expression analysis and UpSet plots above). We used the differentially expressed genes from the author’s analysis of the cortical biopsy dataset (Gazestani et al.^29^). Only positively differentially expressed genes were used. The hypergeometric test was run on intersecting genes using the dhyper function from the Stats R package (v.3.6.3). The background number of genes for each dataset was calculated based on genes expressed in more than 1% of cells.

### Hypergeometric test comparing mouse and iMGL DAM gene expression

Hypergeometric test was performed as above, with the following exceptions. Mouse DAM genes were identified from a published dataset^6^, and mouse microglia background genes were determined from the same dataset as genes expressed in more than 1% of cells. Only positively differentially expressed genes and those with a fold change greater than 0.2 were used (matching our fold change threshold). To compare the background and DAM genes across datasets, the babelgene R package (v.2.9) was used (https://github.com/igordot/babelgene). The hypergeometric test was run on intersecting genes using the dhyper function from the Stats R package (v.3.6.3).

### Cluster occupancy test

To test the statistical significance of the coclustering of iMGL_2, iMGL_8 and human DAMs (BB_GPNMB_LPL), a binomial test was used to determine whether cells from each group were enriched in joint clusters 8 and 15 relative to other clusters. This test used raw numbers rather than percentages. The binom.test function from the Stats R package (v.3.6.3) was used for this analysis.

### SCENIC transcription factor analysis

The post-QC, normalized iMGL gene expression matrix was used as a starting point for analysis with SCENIC^36^ v.1.1.2-2. Cells from the same LIGER cluster were randomly averaged into pseudocells of 20, an approach that has previously been shown to yield more robust results^62^. GENIE3 (v.1.4.3) was used to identity transcription factor–gene coexpression networks, followed by regulon analysis (runSCENIC_2_createRegulons) and regulon scoring per cell (runSCENIC_3_scoreCells) to create a regulon expression matrix for all cells. For the final list of significant regulons, a differential expression test was performed with the presto R package (v.1, wilcoxauc function) (https://github.com/immunogenomics/presto), and results were filtered by adjusted *P* < 0.01 and area under the curve >0.6. Regulons were scaled and centered for plotting using the pheatmap package (https://github.com/raivokolde/pheatmap).

### Lentivirus-exposed versus control iMGLs

Sequencing data were processed with Cell Ranger 3.1.0 (10x Genomics). Fastq files were mapped to a modified reference genome (GRCh38-2020-A/GENCODE v32/Ensembl 98) containing the mCherry gene. Count data were obtained using Cell Ranger’s count function. The count data were further analyzed using Seurat 3.2.2 (ref. ^56^). Count matrices from four conditions were merged, filtered (only cells with 2,000–7,000 genes, 500–60,000 reads and less than 20% mitochondrial RNA were kept) and normalized using standard log-normalization. Integration was performed using the FindIntegrationAnchors and IntegrateData functions. The CellCycleScoring function was used to analyze the number of cycling cells. To analyze the remaining gene expression changes in the dataset, cycling cells were removed (only cells in G1 phase were kept), the data were aggregated and differentially expressed genes were identified using the Deseq2 1.30.1 package^63^ with default parameters (except for adjusted *P* = 0.01). Enriched gene ontology terms were annotated with the enrichR package using database GO_Biological_Process_2015 (ref. ^64^).

### Analysis of iPSC-derived iMGLs and dataset integration

For analysis of lines CW50118, CW500036 and CW70437, single-cell gene expression matrices were first filtered for low-quality cells. As above, we only considered cells with the following characteristics: (1) number of genes: 2,000–7,000; (2) number of UMIs: 500–6,000, (3) percentage mitochondrial RNA 0–0.2%. After this cell-filtration step, cells were merged with the post-QC CNS-substrate-exposed H1 iMGL scRNA-seq dataset above (Fig. 1). To integrate the two replicates, we considered the H1 iMGL replicate (1 or 2) and the iPSC stem cell line as the LIGER dataset variable and applied the LIGER pipeline for dataset integration (*k* = 20, lambda = 6). Clustering was performed using the louvainCluster function (clustering resolution = 0.3). A UMAP embedding was calculated using LIGER’s runUMAP function. The alignment was evaluated with the CalcAlignment LIGER function. Two small clusters were enriched for neuronal genes and specific to the AN-treated conditions for all lines. These were considered to be doublets and removed before visualization and downstream analyses. In addition, a small population of cells enriched for the ExAM module score were removed. All gene expression, metagene and violin plots were produced using LIGER functions and modified using ggplot2.

### Module score iMGL state markers

To examine the similarity of H1-derived and iPSC-derived iMGL states, module scores for each H1 iMGL cluster (Fig. 1) were created. These module scores were calculated for each cell in the iPSC-derived iMGL dataset only using positive differentially expressed genes identified above for each cluster in the H1-derived iMGL dataset (see Single-cell differential expression analysis and UpSet plots). In all cases, the module score was implemented in Seurat using the AddModuleScore function with a control size of 100.

### Bulk RNA-seq analysis

Reads were aligned to the human genome (hg19) using Picard (https://broadinstitute.github.io/picard/). Read counts were obtained using Featurecounts^65^, ComBat-seq was used to adjust for batch effects^66^, genes with fewer than 50 read counts across samples were removed from analyses and DESeq2 was used for differential expression^63^ with Wald test adjusted *P* < 0.05. For the hypergeometric test, the background number of genes for each dataset was calculated based on genes expressed in more than 1% of cells (13,193 genes).

### ATAC-seq data processing

Reads were aligned to the human reference genome (hg19) with the BWA aligner^67^. Duplicates were removed using Picard tools (MarkDuplicates) (https://broadinstitute.github.io/picard/), and peaks were called using HOMER 4.11.1 (ref. ^35^) in ‘histone’ mode. Diffbind^68^ was used to identify differentially represented regions between conditions.

### Statistics and reproducibility

Each experiment was done on multiple independent differentiations and all conditions were included in each differentiation. Sample sizes were based on standards in the field, and experimental treatment groups were randomly assigned.

### Reporting summary

Further information on research design is available in the Nature Portfolio Reporting Summary linked to this article.

## Online content

Any methods, additional references, Nature Portfolio reporting summaries, source data, extended data, supplementary information, acknowledgements, peer review information; details of author contributions and competing interests; and statements of data and code availability are available at 10.1038/s41590-023-01558-2.

## Supplementary information

Reporting Summary

Supplementary Table 1 Mean QC values and number of cells per condition and replicate after QC.

Supplementary Table 2 Differentially expressed genes per cluster for full iMGL dataset (H1).

Supplementary Table 3 Results of differential abundance testing of iMGL clusters across conditions. Adjusted *P* values representing the differential abundance versus control for each substrate exposure for each cluster.

Supplementary Table 4 Shared gene signature in human and iMGL DAMs, hypergeometric test.

Supplementary Table 5 Top 200 shared gene signatures in human and iMGL DAMs, LIGER metagene.

Supplementary Table 6 iMGL ATAC-seq including DAR regions between iMGL ANs and nontreated, list of transcription factors and motifs from HOMER analysis and overlap between HOMER and SCENIC analysis.

Supplementary Table 7 Differentially expressed iMGL regulons identified by SCENIC.

Supplementary Table 8 Differentially expressed genes between control and lentivirus treated iMGLs, significant differences are listed as threshold = TRUE.

Supplementary Table 9 MITF bulk RNA sequencing data, contains DEG analysis and overlap between MITF upregulated genes and microglia states and statistical analysis.

Supplementary Table 10 iPSC line information, also includes scRNA-seq summary data: mean QC values and numbers of cells per condition per line after filtration for iPSC-differentiated iMGLs.

## Data Availability

All iMGL data have been deposited on Terra, including raw and Cell Ranger outputs of iMGL (H1 and CW50118, CW500036 and CW70437) scRNA-seq, fastq and bam files of iMGL untreated and treated with ANs for ATAC-seq, and fastq and bam files of MITF-overexpression and mCherry control bulk RNA sequencing. Summary level data are available at https://app.terra.bio/#workspaces/Stevenslab/public_iMGLdatasets. Raw data are available via managed access at DUOS (https://duos.org); ID: DUOS-000151. Any additional data are available from the corresponding authors.

## Code availability

Code is available from the corresponding authors.
