# Supplementary material for: Exposure of iPSC-derived human microglia to brain substrates enables the generation and manipulation of diverse transcriptional states in vitro
Source: Nat Immunol. 2023 Jul 27;24(8):1382–90. doi: 10.1038/s41590-023-01558-2 (PMC10382323; doi:10.1038/s41590-023-01558-2)
Supplement: Supplementary file 1 — Reporting Summary [file 41590_2023_1558_MOESM1_ESM.pdf]

nature portfolio

Corresponding author(s): Beth Stevens

Last updated by author(s): Jan 9, 2023

Reporting Summary

Nature Portfolio wishes to improve the reproducibility of the work that we publish. This form provides structure for consistency and transparency in reporting. For further information on Nature Portfolio policies, see our Editorial Policies and the Editorial Policy Checklist.

Statistics

For all statistical analyses, confirm that the following items are present in the figure legend, table legend, main text, or Methods section.

n/a	Confirmed	
☐	☒	The exact sample size (*n*) for each experimental group/condition, given as a discrete number and unit of measurement
☐	☒	A statement on whether measurements were taken from distinct samples or whether the same sample was measured repeatedly
☐	☒	The statistical test(s) used AND whether they are one- or two-sided<br>*Only common tests should be described solely by name; describe more complex techniques in the Methods section.*
☐	☒	A description of all covariates tested
☐	☒	A description of any assumptions or corrections, such as tests of normality and adjustment for multiple comparisons
☐	☒	A full description of the statistical parameters including central tendency (e.g. means) or other basic estimates (e.g. regression coefficient) AND variation (e.g. standard deviation) or associated estimates of uncertainty (e.g. confidence intervals)
☐	☒	For null hypothesis testing, the test statistic (e.g. *F*, *t*, *r*) with confidence intervals, effect sizes, degrees of freedom and *P* value noted<br>*Give P values as exact values whenever suitable.*
☒	☐	For Bayesian analysis, information on the choice of priors and Markov chain Monte Carlo settings
☒	☐	For hierarchical and complex designs, identification of the appropriate level for tests and full reporting of outcomes
☒	☐	Estimates of effect sizes (e.g. Cohen's *d*, Pearson's *r*), indicating how they were calculated

Our web collection on statistics for biologists contains articles on many of the points above.

Software and code

Policy information about availability of computer code

Data collection	Data was aquired using the following softwares:<br>qRTPCR software BioRad CFX Manager 3.1<br>flow cytometry: CytExpert 2.5.0.77<br>imaging: NIS elements 4.4, Perkin Elmer Harmony V4.9
Data analysis	data analysis was done using standard code and details description and parameters used can be found in the Methods section of the manuscript. packages used are listed below<br>flow cytometry analysis: FloJo V9<br>Image analysis: FIJI2.3.0, Perkin Elmer Harmony V4.9<br>statistics: Prism v9<br>qRT-PCR analysis CFX Maestro software (BioRad)<br>single cell analysis: Cellranger (version 3.1, 10X Genomics), LIGER (version 0.5), Seurat (either version 2.3.4 or 3.2.1), MAST package, DirichletReg R package, fgsea package, Monocle3, SCENIC3, ComplexHeatmap R package, “UpSetR” R package, Stats R package, babelgene R package, presto R package, enrichR, DESEQ2, Picard,<br>bulk RNA-seq:Featurecounts2.0.3, ComBat-seq3.36.0, DESeq21.28.1<br>ATAC-seq: Picard tools, HOMER 4.11.1, Diffbind 2.16.2

For manuscripts utilizing custom algorithms or software that are central to the research but not yet described in published literature, software must be made available to editors and reviewers. We strongly encourage code deposition in a community repository (e.g. GitHub). See the Nature Portfolio guidelines for submitting code & software for further information.

Data

Policy information about availability of data

All manuscripts must include a data availability statement. This statement should provide the following information, where applicable:

- Accession codes, unique identifiers, or web links for publicly available datasets
- A description of any restrictions on data availability
- For clinical datasets or third party data, please ensure that the statement adheres to our policy

All iMGL data is deposited on Terra, including raw and Cell Ranger output of iMGL (H1 and CW50118, CW500036 and CW70437) single cell RNA-sequencing, fastq and bam files of iMGL untreated and treated with apoptotic neurons for ATAC-seq and fastq and bam files of MITF overexpressing and mCherry control bulk RNA-sequencing. Raw data is available via managed access at DUOS https://www.duos.org; ID: DUOS-000151. Any additional data and code is available from the corresponding authors. Terra link: https://app.terra.bio/#workspaces/Stevenslab/public_iMGLdatasets

Human research participants

Policy information about studies involving human research participants and Sex and Gender in Research.

Reporting on sex and gender	NA
Population characteristics	NA
Recruitment	NA
Ethics oversight	NA

Note that full information on the approval of the study protocol must also be provided in the manuscript.

Field-specific reporting

Please select the one below that is the best fit for your research. If you are not sure, read the appropriate sections before making your selection.

☒ Life sciences ☐ Behavioural & social sciences ☐ Ecological, evolutionary & environmental sciences

For a reference copy of the document with all sections, see nature.com/documents/nr-reporting-summary-flat.pdf

Life sciences study design

All studies must disclose on these points even when the disclosure is negative.

Sample size	For single cell analysis , ATAC-sseq and bulk RNA-seq , sample size was determine based on standards in the field (see PMID: 35953545 for example) . For immunocytochemistry and in situ hybridization at least 50 cells were measured across 2 biological replicates for each conditions. For qRT-PCR, at least 4 biological replicates were done for each conditions. All experiments were done across multiple differentiations and all conditions were done on each differentiation.
Data exclusions	No data was discarded except for data filtered during dataset quality control (applies to all single-cell/single-nuclei experiments), which was performed according to field standard techniques as described in methods.<br>single cell exclusion criteria 1) Number of Genes: 2000-7000, 2) Number of UMIs: 500-6000, 3) Percentage mitochondrial RNA 0-0.2%.
Replication	replication was performed for all experiments using at least 2 biological replicates. All experiments were done across multiple differentiations, all conditions were done on each differentiation, all attempt to replicate the data were succesfull
Randomization	for all experiments, all experimental groups were done simultaneously. Samples were randomly allocated in experimental group
Blinding	Investigators were not blinded. All analysis were applied to all samples without adjustment for conditions or genotypes

Reporting for specific materials, systems and methods

We require information from authors about some types of materials, experimental systems and methods used in many studies. Here, indicate whether each material, system or method listed is relevant to your study. If you are not sure if a list item applies to your research, read the appropriate section before selecting a response.

Materials & experimental systems

n/a	Involved in the study
☐	☒ Antibodies
☐	☒ Eukaryotic cell lines
☒	☐ Palaeontology and archaeology
☐	☒ Animals and other organisms
☒	☐ Clinical data
☒	☐ Dual use research of concern

Methods

n/a	Involved in the study
☒	☐ ChIP-seq
☐	☒ Flow cytometry
☒	☐ MRI-based neuroimaging

Antibodies

Antibodies used

flow cytometry: CD45-FITC (BioLegend #368508), CD11B-APC-750 (BioLegend #982616), P2RY12-PB450 (BioLegend #392105), Cx3CR1-PrCp (BioLegend #341614)
ICC: TREM2 (R&D systems, AF1828-SP) 1/100, GPNMB (Cell Signaling, E1YZJ), Donkey anti-Rabbit 488 (Thermo Fisher Scientific) 1/500, Goat anti-Rabbit 594 (Thermo Fisher Scientific) 1/500, APOE clone 6B9 (Helmholtz Antibody Collection) (1/100)

Validation

APOE validation can be found https://www.alzforum.org/alzantibodies/apoe-clone-6b9

Biolegends and TREM2 antibodies were validated by the manufacturer and used previously in PMID: 28426964
Biolegend antibodies validation from manufacturers: To ensure they are both specific and sensitive, we validate our antibodies through a variety of methods including:Testing on multiple cell and tissue types with a variety of known expression levels.Validation in multiple applications as a cross-check for specificity and to provide additional clarity for researchers. Comparison to existing antibody clones.
Using cell treatments to modulate target expression, such as phosphatase treatment to ensure phospho-antibody specificity.

Cells snailing antibodies were previously validated by the manufacturer
Cell signaling : To ensure our antibodies will work in your experiment, we adhere to the Hallmarks of Antibody Validation™, six complementary strategies that can be used to determine the functionality, specificity, and sensitivity of an antibody in any given assay. CST adapted the work by Uhlen, et. al., ("A Proposal for Validation of Antibodies." Nature Methods (2016)) to build the Hallmarks of Antibody Validation, based on our decades of experience as an antibody manufacturer and our dedication to reproducible science.

Eukaryotic cell lines

Policy information about cell lines and Sex and Gender in Research

Cell line source(s)

The following iPSC lines used in Figure 6 and Figure S12 were obtained from the CIRM hPSC Repository funded by the California Institute of Regenerative Medicine (CIRM): CW50118, CW50008, CW50065, CW500036 and CW70437.
H1 embryonic stem cell line was obtained from WiCell
The TREM2 knockout and isogenic control iPSC lines were previously characterized(McQuade et al. 2020). These lines were derived from cell line AICS-0036-006 from the NIGMS Human Genetic Cell Repository at the Coriell Institute for Medical Research
HEK293T cells were purchased from ATCC

Authentication

All cell lines were authenticated in 2020 using genotyping analysis (Fluidigm FPV5) performed at the Broad Institute

Mycoplasma contamination

all iPSC and iMGL cultures are routinely tested for mycoplasma contamination using MycoAlert Mycoplasma detection kit from Lonza (LT07-118) and tested negative

Commonly misidentified lines
(See ICLAC register)

no misidentified lines were used in this study

Animals and other research organisms

Policy information about studies involving animals; ARRIVE guidelines recommended for reporting animal research, and Sex and Gender in Research

Laboratory animals

C57/Bl6 older than 28 days but no older than 90 days were used in this

Wild animals

NA

Reporting on sex

NA

Field-collected samples

All experiments were approved by the Broad Institute IACUC (Institutional Animal Care and Use Committee) and conducted in

Field-collected samples	accordance with the NIH Guide for the Care and Use of Laboratory Animals. Mice were housed at AAALAC-approved facilities on a 12-hour light/dark cycle, with food and water available ad libitum
Ethics oversight	Broad Institute IACUC (Institutional Animal Care and Use Committee)

Note that full information on the approval of the study protocol must also be provided in the manuscript.

Flow Cytometry

Plots

Confirm that:

☒ The axis labels state the marker and fluorochrome used (e.g. CD4-FITC).

☒ The axis scales are clearly visible. Include numbers along axes only for bottom left plot of group (a 'group' is an analysis of identical markers).

☒ All plots are contour plots with outliers or pseudocolor plots.

☒ A numerical value for number of cells or percentage (with statistics) is provided.

Methodology

Sample preparation	iMGLs were detached using cold PBS, then resuspended in FACS buffer (PBS containing 2%BSA and 0.05mM EDTA). Samples were incubated for 15 mins in human Fc block (BD Biosciences) followed by 1h staining with conjugated antibodies (see below) at 4C. Samples were washed 3x with FACS buffer and resuspended in 500ul of FACS buffer for flow cytometry
Instrument	CytoFLEX S analyzer (Beckman Coulter)
Software	All analyses were performed using FlowJo V10 and statistical analysis performed using Prism9.
Cell population abundance	shown in each Figure S1A, S3B
Gating strategy	all flow cytometry experiments (S1A-B, S3A) were gated based on cell vs debris (FCS-A vs SSC-A), singlets (FSC-A vs FSC-H), live (DAPI negative), specific gatings for antibodies was based on negative unstained sample and for phagocytosis sample without phagocytosis substrate.

☒ Tick this box to confirm that a figure exemplifying the gating strategy is provided in the Supplementary Information.
